# Histone N-terminal acetyltransferase NAA40 links one-carbon metabolism to chemoresistance

**DOI:** 10.1038/s41388-021-02113-9

**Published:** 2021-11-16

**Authors:** Christina Demetriadou, Anastasia Raoukka, Evelina Charidemou, Constantine Mylonas, Christina Michael, Swati Parekh, Costas Koufaris, Paris Skourides, Panagiotis Papageorgis, Peter Tessarz, Antonis Kirmizis

**Affiliations:** 1 Department of Biological Sciences, University of Cyprus, 2109 Nicosia, Cyprus; 2 Max Planck Institute for Biology of Ageing, Cologne, Germany; 3 Department of Life Sciences, European University Cyprus, 2404 Nicosia, Cyprus; 4 Cologne Excellence Cluster on Stress Responses in ageing-associated Diseases (CECAD), Joseph-Stelzmann-Str. 26, 50931 Cologne, Germany

**Keywords:** Cancer metabolism, Colorectal cancer, Histone post-translational modifications, Transcription

## Abstract

Aberrant function of epigenetic modifiers plays an important role not only in the progression of cancer but also the development of drug resistance. N-alpha-acetyltransferase 40 (NAA40) is a highly specific epigenetic enzyme catalyzing the transfer of an acetyl moiety at the N-terminal end of histones H4 and H2A. Recent studies have illustrated the essential oncogenic role of NAA40 in various cancer types but its role in chemoresistance remains unclear. Here, using transcriptomic followed by metabolomic analysis in colorectal cancer (CRC) cells, we demonstrate that NAA40 controls key one-carbon metabolic genes and corresponding metabolites. In particular, through its acetyltransferase activity NAA40 regulates the methionine cycle thereby affecting global histone methylation and CRC cell survival. Importantly, NAA40-mediated metabolic rewiring promotes resistance of CRC cells to antimetabolite chemotherapy in vitro and in xenograft models. Specifically, NAA40 stimulates transcription of the one-carbon metabolic gene thymidylate synthase (*TYMS*), whose product is targeted by 5-fluorouracil (5-FU) and accordingly in primary CRC tumours *NAA40* expression associates with *TYMS* levels and poorer 5-FU response. Mechanistically, NAA40 activates *TYMS* by preventing enrichment of repressive H2A/H4S1ph at the nuclear periphery. Overall, these findings define a novel regulatory link between epigenetics and cellular metabolism mediated by NAA40, which is harnessed by cancer cells to evade chemotherapy.

## Introduction

Histone acetyltransferases (HATs) play a pivotal role in the regulation of gene transcription and chromatin structure by transferring an acetyl group from acetyl-CoA to either the side chain of internal lysine residues or the N-terminal tip of histone proteins [1]. Several studies have linked aberrant histone lysine acetylation with cancer progression and chemoresistance, suggesting that HAT enzymes could be attractive therapeutic targets [2]. N-alpha-acetyltransferase 40 (NAA40), a member of the N-terminal acetyltransferase (NAT) family of enzymes, serves as a highly selective HAT by acetylating specifically the alpha-amino group of serine 1 on histones H4 (N-acH4) and H2A (N-acH2A) [3]. A recent multi-omic analysis revealed that NAA40 is upregulated in a diverse range of tumours and correlates with poor overall survival of cancer patients [4]. In addition, accumulating evidence derived from in vitro, in vivo and clinical studies by our group and others implicate NAA40 and its associated histone N-terminal acetylation in tumour growth and metastasis of different cancers including lung, liver and colorectal cancer (CRC) [5–7]. Despite its importance in cancer development and metastasis, the role of this epigenetic modifier in cancer chemotherapy response remains elusive.

In order for tumour cells to acquire and maintain unabated cell proliferation and chemoresistance they need to rewire their metabolic program favoring core functions like nucleotide biosynthesis and energy production [8]. Emerging evidence demonstrate that metabolism dynamically communicates with the cellular epigenetic machinery influencing one another [9]. In fact, epigenetic rewiring during malignant transformation enables histone modifiers and their mediated histone marks to directly affect metabolic gene expression. Reciprocally, hijacking the metabolic network influences the availability of crucial intermediate metabolites such as acetyl-coenzyme A (ac-CoA) and S-adenosylmethionine (SAM), which serve as substrates of chromatin-modifying enzymes thus impacting epigenetic modifications [10]. Although various studies so far showed that metabolic rewiring contributes to epigenetic alterations in cancer cells conferring enhanced growth and metastatic potential, far less is known about the regulation of metabolism by chromatin-modifying factors in controlling anti-cancer drug therapy.

In this study, we found that NAA40 regulates the expression of genes encoding vital enzymes involved in one-carbon (1 C) metabolic network thereby influencing the abundance of intermediary metabolites of this pathway including S-adenosyl methionine (SAM) and uridine monophosphate (UMP). As a result, NAA40 depletion induces global histone methylation levels and attenuates CRC cell survival. Notably, NAA40-mediated activation of the 1C-metabolic gene *TYMS* confers 5-FU resistance to CRC cells and in human colorectal tumours NAA40 expression positively correlates with *TYMS* levels and worse response of patients to 5-FU-based chemotherapy. At the molecular level, we show that NAA40 stimulates transcription of *TYMS* by controlling the spatial distribution of its antagonistic histone mark H2A/H4S1ph within the nucleus. Collectively, these findings reveal NAA40 as novel regulator of cancer cell metabolism and provide new insight for predicting or overcoming therapy resistance in colorectal cancer.

## Results

### NAA40 regulates one-carbon metabolism in colorectal cancer cells

**Fig. 1:**
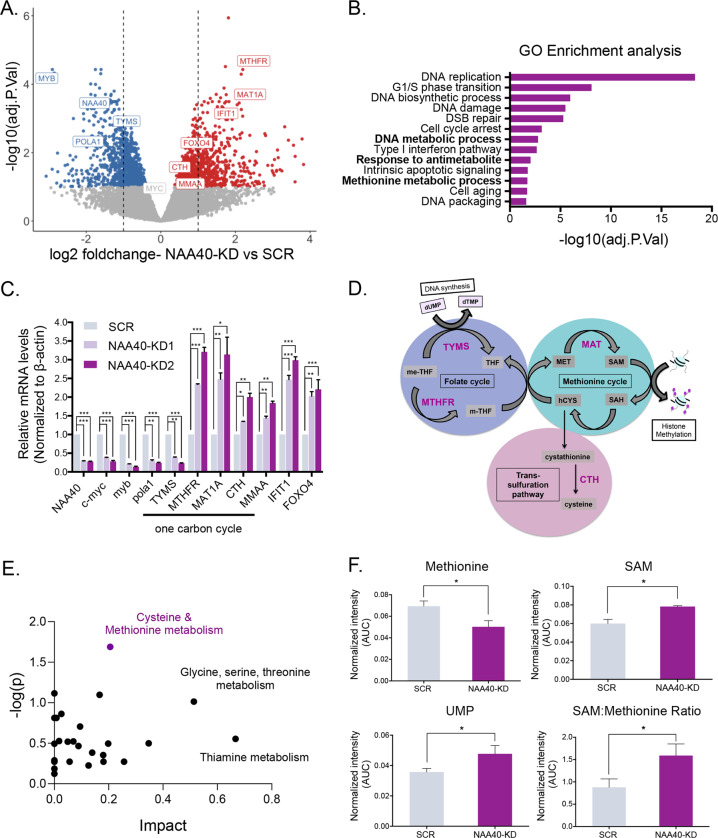
NAA40 regulates the levels of key 1 C metabolic genes and their associated metabolites. **A** Volcano plot comparing mRNA levels between NAA40 depleted (NAA40-KD2) and SCR control HCT116 cells as determined by RNA-seq analysis. Upregulated genes upon loss of NAA40 are shown in red (adjusted *P* < 0.01 and logFC > 1) and downregulated genes in blue (adjusted *P* < 0.01 and logFC < −1). **B** Gene ontology analysis of all differentially expressed genes showing enriched biological processes following depletion of NAA40. **C** Quantitative real-time PCR (qRT-PCR) analysis (mean ± s.d., *N* = 3) of the indicated genes normalized to *β-actin* mRNA levels in dox-treated SCR and two independent NAA40-KD lines (NAA40-KD1 and NAA40-KD2) in HCT116 cells. **D** Schematic of the folate, methionine and trans-sulfuration cycles of the one-carbon metabolic network depicting key enzymes and intermediate metabolites. **E** Enrichment analysis of altered metabolic pathways based on detected metabolites by UPLC-MS in NAA40-KD2 versus SCR control cells. Impact represents the enrichment ratio; the number of total compounds in the pathway divided by the number of hits. **F** Intracellular levels of the indicated one-carbon metabolites or their ratios (mean ± s.e., *N* = 3) in dox-treated SCR and NAA40-KD2 HCT116 cells. All statistical analyses were performed using unpaired two-tailed Student’s *t* test (*p < 0.05, **p < 0.01, ***p < 0.001).

Considering that NAA40 was recently reported to be significantly elevated in colorectal cancer (CRC) tissues and stimulated tumour cell growth in vitro and in vivo [5], we sought to investigate its molecular role in CRC. To do this, we initially conducted RNA-seq analysis using an inducible shRNA-mediated knockdown system that we have previously developed in CRC cells [5]. Comparison of doxycycline treated Scramble (SCR) and NAA40-knockdown (NAA40-KD) HCT116 stable cells revealed that NAA40 depletion leads to altered expression of 2102 genes, with differential expression determined at a threshold of *p* < 0.05 and an absolute log fold change >1 (Fig. 1A). Gene Ontology (GO) analysis of the differentially expressed genes illustrated that loss of NAA40 alters sets of genes involved in cancer-related processes in support of our previous finding [5]. Specifically, some of the most significantly enriched GO terms included DNA replication, DNA damage and repair pathways, as well as cell cycle phase transition (Fig. 1B). In line with these findings, we observed that cells deprived of NAA40 are restricted in the G1/S phase of the cell cycle (Supplementary Fig. S1A), further supporting the tumour promoting role of NAA40. Intriguingly, among the most notably enriched GO terms were ones pointing to a connection with metabolism such as the methionine metabolic pathway, regulation of DNA metabolic process and response to anti-metabolite therapy (Fig. 1B). Key genes within these categories included methylenetetrahydrofolate reductase (*MTHFR*), methionine adenosyltransferase 1 A (*MAT1A*), cystathionine gamma-lyase (*CTH*) and metabolism of cobalamin associated A (*MMAA*) which were significantly upregulated, while thymidylate synthase (*TYMS*) was markedly downregulated in the absence of NAA40 (Fig. 1A). The expression of these and other genes identified during our transcriptomic study was validated through quantitative real time PCR (qRT-PCR) analysis (Fig. 1C).

The above identified differentially expressed genes control important reactions within the 1C-metabolic network, which interconnects the methionine, folate and trans-sulfuration cycles (Fig. 1D), circulating 1C-units to support a multitude of fundamental cellular activities, including nucleotide synthesis and the production of the universal methyl donor SAM [11, 12]. Since NAA40 knockdown influenced the transcription of important one-carbon metabolic genes, we next examined the impact of NAA40 on the metabolome of CRC cells. To address this, we performed targeted metabolomic analysis using a liquid chromatography/mass spectrometry (LC/MS) approach to identify metabolites whose abundance displays significant change in cells devoid of NAA40 compared to doxycycline-treated control cells. In accordance with our transcriptomic data, enrichment analysis of deregulated metabolites in NAA40 depleted cells revealed cysteine and methionine metabolism as the most significantly modulated metabolic pathway (Fig. 1E). Specifically, NAA40 deficiency resulted in a substantial increase of intracellular SAM pools and a smaller increase in the abundance of S-adenosyl-L-homocysteine (SAH), homocysteine, methylene-tetrahydrofolate (me-THF) and glycine intermediary metabolites, whereas methionine levels were significantly lower relative to SCR control cells (Fig. 1F, Supplementary Fig. S1B). These results are consistent with the induction of *MTHFR* and *MAT1A*, and repression of *TYMS* observed in the gene expression analysis (Fig. 1A, C). Notably, NAA40 depletion led to increased SAM/methionine ratio and accumulation of UMP which is the central precursor for thymidine synthesis (Fig. 1F), suggesting that NAA40 depleted cells may therefore possess an enhanced capacity for methylation reactions. In contrast, we do not detect major changes in metabolite levels of other central metabolic pathways, such as glycolysis in these cancer cells, indicating that the effects of NAA40 knockdown are specific towards 1C-metabolism (Supplementary Fig. S1B). Altogether, these findings establish a role for NAA40 histone acetyltransferase in the regulation of one-carbon metabolism in CRC cells.

### Regulation of one-carbon metabolism by NAA40 rewires global histone methylation

**Fig. 2:**
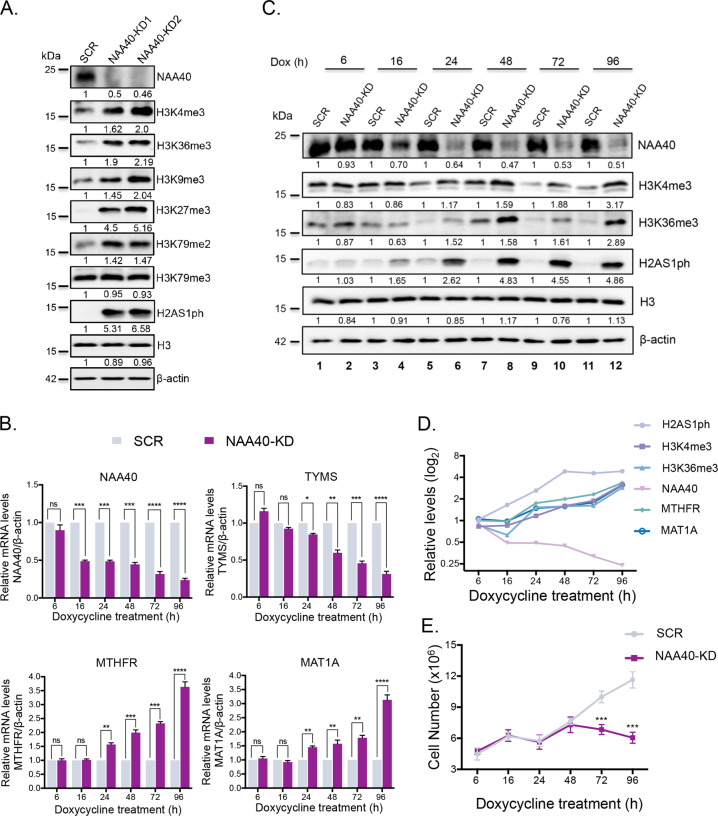
NAA40-mediated changes in one-carbon metabolism impact global histone methylation. **A** Representative western blot analysis (*N* = 3) of cell extracts derived from dox-treated SCR or two independent NAA40-KD (NAA40-KD1 and NAA40-KD2) HCT116 cells lines using the indicated antibodies. The numbers below each blot indicate densitometry analysis of protein levels in NAA40-KD relative to SCR after normalization to the β-actin bands. **B** qRT-PCR analysis (mean ± s.d., *N* = 3) of *NAA40*, *TYMS*, *MTHFR* and *MAT1A* mRNA levels normalized to β-actin performed in SCR or NAA40-KD2 HCT116 cells treated with doxycycline for the indicated time points. **C** Representative western blot analysis (*N* = 3) in proteins extracted at different time points from dox-treated HCT116 SCR and NAA40-KD2 cells using the indicated antibodies. The densitometry numbers below each blot define the normalized levels of each protein against β-actin relative to SCR cells. **D** Relative changes in modification (H2AS1ph, H3K4me3 and H3K36me3) or mRNA (*NAA40*, *MTHFR* and *MAT1A*) levels following NAA40 depletion for the indicated time points. **E** Graph showing the number of HCT116 SCR and NAA40-KD2 cells treated with doxycycline for the indicated time points (mean ± s.d., *N* = 3). All statistical analyses were performed using unpaired two-tailed Student’s *t* test (ns = no significance, *p < 0.05, **p < 0.01, ***p < 0.001, ****p < 0.0001).

Previous studies have linked fluctuations in the availability of the principal methyl donor SAM with bulk changes in chromatin methylation [13–16]. Since NAA40 controls SAM levels, we next sought to investigate whether NAA40 depletion impacts the epigenome by affecting histone methylation levels. Initially, we validated NAA40 depletion through loss of its associated acetyltransferase activity towards histone N-termini by examining the appearance of its previously reported antagonistic phosphorylation mark at serine 1 (Fig. 2A) [6]. This was necessary due to the lack of an antibody detecting histone N-terminal acetylation. Specifically, we found that loss of NAA40 in HCT116 cells dramatically potentiated serine 1 phosphorylation at both histones H2A (H2AS1ph) and H4 (H4S1ph) with the signal at the former being more readily detected (Supplementary Fig. S2A). Once we validated that NAA40 function was efficiently diminished, we then monitored the levels of several histone methylation marks. Remarkably, knockdown of NAA40 results in a robust increase in the total levels of various histone methylation marks associated with transcription, including both permissive (H3K4me3, H3K36me3 and H3K79me2) and repressive (H3K9me3 and H3K27me3) methylations (Fig. 2A, Supplementary Fig. S2B), which is consistent with the detected accumulation in SAM levels (Fig. 1F). However, this increase was not universal since the levels of other methylation marks, such as H3K79me3, remained unaffected. In support of this observation, recent evidence confirms that fluctuations in SAM abundance do not impact all methylated histone sites, but rather directed to specific residues in a context-dependent manner [16–18]. Moreover, subcellular fractionation experiments illustrated that the induction of histone methylation occurs mainly on chromatin-associated histone proteins (Supplementary Fig. S2C).

To gain further insight into NAA40-mediated regulation of one carbon metabolism and its connection with histone methylation changes, we then monitored the temporal dynamics of these alterations. To achieve this, SCR and NAA40-KD cells were treated with doxycycline and examined at various time points (6, 16, 24, 48, 72, 96 h). Significant NAA40 depletion was detected from 16 h after dox treatment and progressively increased based on both NAA40 mRNA (Fig. 2B) and protein levels (Fig. 2C) as well as the appearance of its antagonistic histone mark H2AS1ph (Fig. 2C). Deregulation of one-carbon metabolic genes *TYMS*, *MTHFR* and *MAT1A* followed NAA40 depletion since it was detected at 24 h after dox treatment and progressively increased until 96 h (Fig. 2B). Importantly, this gene induction was concurrent to the rising levels of H3K4me3 and H3K36me3 (Fig. 2C). Comparison of all these alterations clearly indicates that the expression changes in one-carbon metabolic genes occur shortly after NAA40 depletion and coincide temporally with the histone methylation changes (Fig. 2D). Because we have previously reported that NAA40-knockdown results in reduced viability of CRC cells [5], we wanted to exclude the possibility that the observed coordinated changes in metabolic gene expression and histone methylation are occurring as a response to growth retardation signals. We found that these chromatin changes precede the effects on cell survival, since reduction in cell growth and viability were only apparent after 72 h of NAA40 depletion (Fig. 2E, Supplementary Fig. S3). These findings overall show that the effects of NAA40 knockdown on metabolic rewiring and its corresponding epigenome changes are not prompted by a cell growth defect and suggest that NAA40-dependent transcriptional effects in one-carbon metabolism drive histone methylation changes.

Given that NAA40 mainly acetylates histone proteins co-translationally we next wondered if the effects of NAA40 depletion are depended on active cell cycle. Intriguingly, transcriptomic analysis in synchronized primary human fibroblast cells showed that NAA40 expression levels are markedly induced during S phase, when histone proteins are mainly expressed and synthesized (Supplementary Fig. S4A). To examine whether an active cell cycle is required for the NAA40-KD effects, we cultured NAA40 expressing (−Dox) and deficient (+Dox) cells under serum starvation conditions (0.5% or 0%), which are known to induce arrest in the G0/G1 phase of the cell cycle [19, 20] (Supplementary Fig. S4B). Although serum starvation alone reduced cell growth and somewhat induced H3K36me3, the lack of NAA40 in combination with serum starvation further enhanced the cell growth defect and the induction of histone methylation (Supplementary Fig. S4B,C). Consistently, doxycycline-treated NAA40-KD cells that were serum starved exhibited reduced levels of *TYMS* and increased expression of *MTHFR*, which is similar to serum-rich (10%) NAA40-depleted cells albeit to a lesser extent (Supplementary Fig. S4D). These findings demonstrate that the effects of NAA40 depletion occur independently of an active cell cycle.

**Fig. 3:**
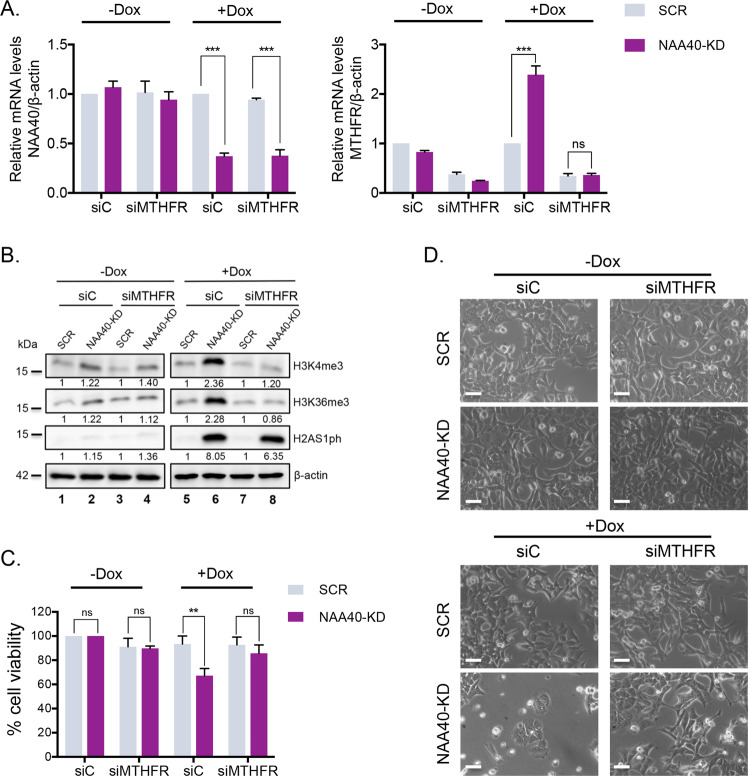
The effect of NAA40 on histone methylation is driven through one-carbon metabolism. **A** qRT-PCR analysis (mean ± s.d., *N* = 3) monitoring *NAA40* (left panel) and *MTHFR* (*right panel*) mRNA levels normalized to *β-actin* of HCT116 SCR or NAA40-KD2 cells transiently transfected with MTHFR-specific (siMTHFR) or control (siC) siRNAs in the presence or absence of doxycycline treatment. **B** Representative western blot analysis (*N* = 3) of proteins extracted from siC or siMTHFR transfected SCR and NAA40-KD2 HCT116 cells treated with or without doxycycline using the indicated antibodies. The values below each blot were calculated by densitometry analysis of each protein band relative to the corresponding SCR control after normalization with β-actin. **C** MTT assay (mean ± s.d., *N* = 3) in dox-treated or untreated SCR and NAA40-KD2 cells that were transiently transfected with siC or siMTHFR. **D** Phase contrast microscopy of dox-inducible HCT116 SCR and NAA40-KD2 cells transfected with siC or siMTHFR. Cells were captured in at least five fields of view (100X magnification). The images are representative fields from at least three independent replicates. Scale bar, 100 μm. All statistical analyses were performed using unpaired two-tailed Student’s *t* test (ns = no significance, **p < 0.01, ***p < 0.001).

To support the above notion that NAA40 driven metabolic gene expression changes are responsible for the increased histone methylation phenotype, we next depleted MTHFR using siRNA transfection experiments to attenuate one-carbon metabolism (Fig. 3A). Unlike the control siRNA which had no effect (Fig. 3B–D), MTHFR siRNA specifically prevented the induction of histone methylation (Fig. 3B, compare lanes 7 and 8 with lanes 5 and 6), and rescued the cell viability and growth defect caused by NAA40 depletion (Fig. 3C, D bottom images). To ensure that MTHFR is acting downstream of NAA40, we repeated these experiments in SCR and NAA40-KD cells without dox treatment which maintain *NAA40* expression (Fig. 3A, left plot). As expected, *MTHFR* was again depleted (Fig. 3A, right plot) but we did not detect any dramatic changes in either histone methylation or CRC cell growth and viability (Fig. 3B, compare lanes 3 and 4 with lanes 1 and 2, Fig. 3C, and Fig. 3D top images). These results reinforce the idea that the effects on histone methylation levels are dependent on NAA40-mediated regulation of one-carbon metabolism and may impinge on CRC cell survival.

Finally, to examine whether the effects of NAA40 on histone methylation occur universally in colon cancer cells we examined three additional CRC cell lines (HT-29, SW480 and SW620). In line with the results observed in HCT116 cells, the bulk levels of histone methylation were elevated in all three different cell lines upon NAA40 knockdown (Supplementary Fig. S5A). The increase in histone methylation seen in doxycycline treated NAA40-KD cells was accompanied by transcriptional upregulation of the one-carbon metabolic gene *MTHFR* (Supplementary Fig. S5B) and consistently the viability of these cells declines in the absence of NAA40 [5]. Collectively, these data suggest that in colorectal cancer cells NAA40-mediated regulation of one-carbon metabolic gene expression controls the global levels of different histone methylation marks.

### NAA40 regulates one-carbon metabolism through its histone acetyltransferase activity

**Fig. 4:**
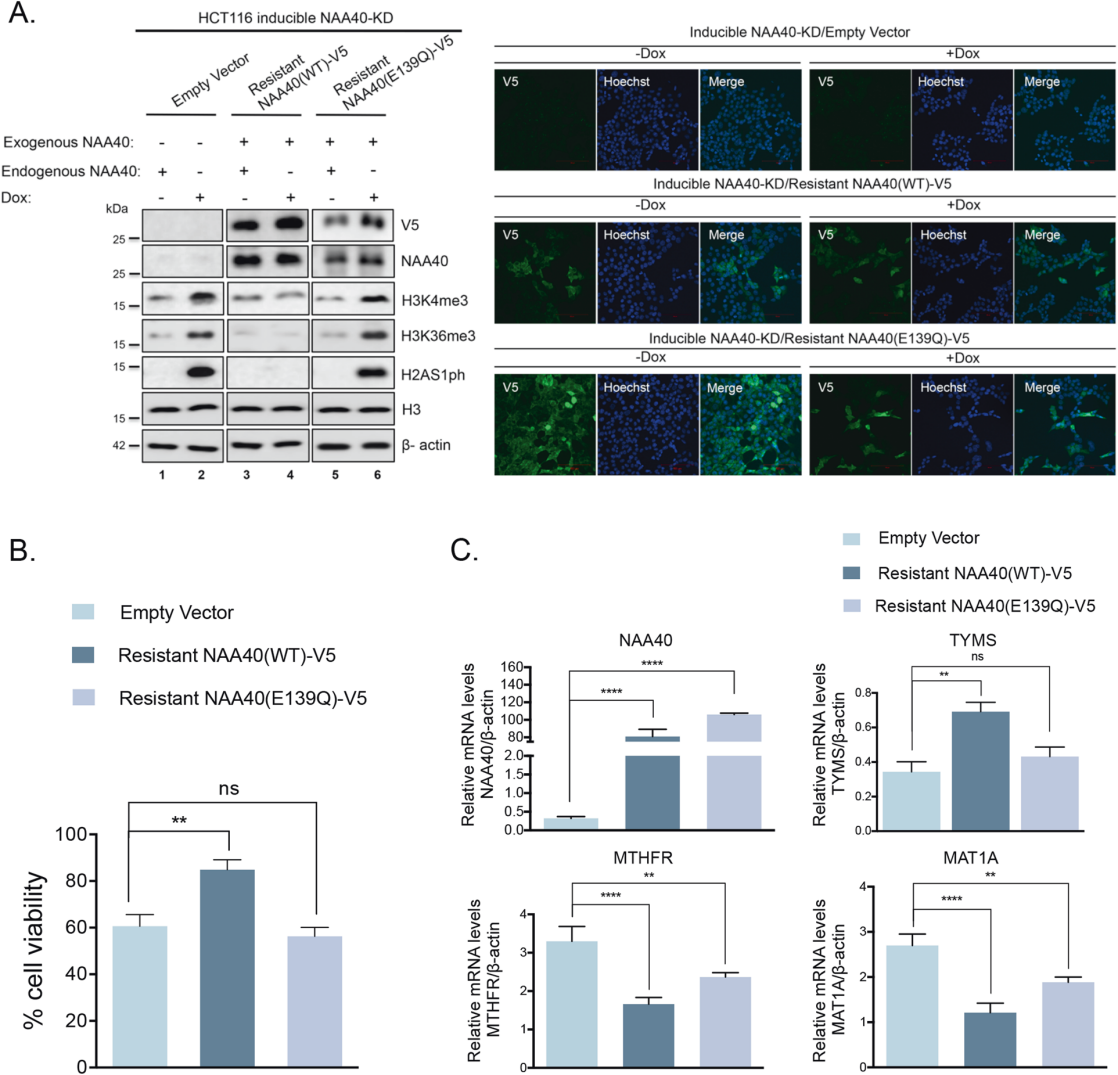
NAA40-mediated regulation of one-carbon metabolism, histone methylation and cell viability are dependent on its acetyltransferase activity. **A** Western blot analysis (*N* = 3) of HCT116 inducible NAA40-KD cells transduced with Empty vector, shRNA-Resistant NAA40(WT)-V5 or shRNA-Resistant NAA40(E139Q)-V5 plasmids and treated with or without doxycycline using antibodies against the specified antibodies (left panel). Representative confocal images of V5-tag (green) or Hoechst (blue) in the indicated stable cell lines treated with or without doxycycline (right panel). Scale bar, 100 μm. **B** MTT assay (mean ± s.d., *N* = 3) to assess cell viability in dox-treated NAA40-KD2 cells expressing the indicated proteins. **C** qRT-PCR analysis (mean ± s.d., *N* = 3) demonstrating the mRNA levels of *NAA40*, *TYMS*, *MTHFR* and *MAT1A* normalized to *β-actin* in HCT116 inducible NAA40-KD cells transduced with the indicated plasmids in the presence or absence of dox treatment. All statistical analyses were performed using unpaired two-tailed Student’s *t* test (ns = no significance, *p < 0.05, **p < 0.01, ****p < 0.0001).

Next, we sought to investigate whether the above described NAA40-dependent outcomes are indeed specific to the loss of NAA40 and mediated through its acetyltransferase activity, reported to act selectively on histones [21]. To this end, we devised RNAi-rescue experiments by engineering doxycycline-inducible NAA40-KD cells that ectopically express either a wild type NAA40-V5 mRNA that was resistant to shRNA-mediated depletion (Resistant NAA40(WT)-V5), or a resistant catalytically inactive version of NAA40-V5 (Resistant NAA40(E139Q)-V5) [3, 21]. We initially validated our engineered system showing that the shRNA-resistant NAA40(WT)-V5 cells maintain expression of exogenous NAA40(WT)-V5 under dox treatment and as a result there is no accumulation of the opposing mark H2AS1ph, showing that the exogenous NAA40(WT)-V5 could complement the function of the endogenous NAA40 enzyme (Fig. 4A, compare lane 3 with 4). Most importantly, the exogenous shRNA-resistant catalytically inactive NAA40(E139Q)-V5 remains unchanged under dox treatment, but the antagonistic mark H2AS1ph accumulates indicating that this inactive version of NAA40 is unable to modify histone N-termini and thus cannot complement the catalytic function of the endogenous enzyme (Fig. 4A, compare lane 5 with 6). In addition to immunoblot analysis, the expression and localization of the exogenous wild-type or catalytically-inactive NAA40 protein in the presence or absence of doxycycline were validated using confocal microscopy (Fig. 4A, right panel). Consistent with its previously reported cellular localization [3], the exogenous NAA40 protein is localized both in the cytosol and the nucleus.

Having validated this system, we then examined how the levels of histone methyl marks and cell viability are affected in these various engineered cells. Consistent with the above results, the increase of H3K4me3 and H3K36me3 was again detected upon depletion of endogenous NAA40 (Fig. 4A, lanes 1–2). However, this histone methylation enhancement was rescued by exogenous expression of the resistant NAA40(WT)-V5 (Fig. 4A, lanes 3–4), reinforcing the notion that this effect is mediated by specific loss of NAA40. Of note, the shRNA-resistant enzymatically inactive NAA40(E139Q)-V5 was unable to reduce the levels of H3K4me3 and H3K36me3 under dox treatment suggesting that these effects are driven by the acetyltransferase activity of NAA40 (Fig. 4A, lanes 5–6). Accordingly, the resistant NAA40(WT)-V5 protein rescued cell viability almost fully while the catalytically dead NAA40(E139Q)-V5 failed to restore CRC cell viability in the absence of the endogenous enzyme showing once again that this inactive form cannot complement the function of intact NAA40 (Fig. 4B).

Last, we found that overexpression of the shRNA-resistant NAA40(WT)-V5 prevented robust upregulation of *MTHFR* and *MAT1A* as well as downregulation of *TYMS* under dox conditions and this was consistent with the absence of H3K4me3 and H3K36me3 enhancement (compare Fig. 4A, C). Nevertheless, forced expression of the shRNA-resistant catalytically dead NAA40(E139Q)-V5 failed to block *MTHFR* and *MAT1A* induction as well as *TYMS* reduction after depletion of endogenous NAA40 by dox treatment, which was again consistent with the detected increase in histone methylation marks (compare Fig. 4A, C). Altogether, these results demonstrate that the function of NAA40 in regulating one-carbon metabolic gene expression, global histone methylation as well as CRC cell viability is specifically attributed to its acetyltransferase activity.

### Regulation of one-carbon metabolism by NAA40 renders CRC cells resistant to antimetabolite drug 5-FU

**Fig. 5:**
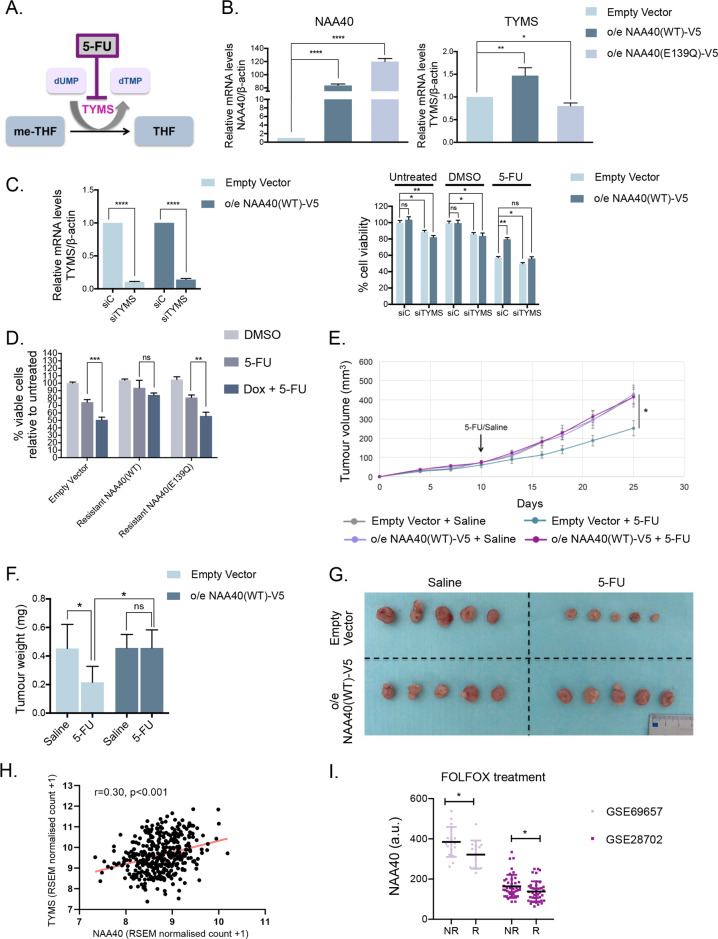
NAA40 confers 5-FU resistance through activation of the one-carbon metabolic gene *TYMS*. **A** Schematic of the metabolic reaction catalyzed by TYMS enzyme which is targeted by 5-FU. **B** qRT-PCR analysis (mean ± s.d., *N* = 3) of the indicated HCT116 overexpression cell lines using specific primers for *NAA40* and *TYMS*. The presented values are normalized to *β-actin* mRNA. **C** qRT-PCR analysis (mean ± s.d., *N* = 3) of *TYMS* mRNA levels normalized to *β-actin* in HCT116 cells stably overexpressing NAA40(WT)-V5 or empty vector that are transiently transfected with control (siC) or TYMS-specific (siTYMS) siRNAs (left panel). MTT assay in HCT116 carrying empty vector control or NAA40(WT)-V5 overexpressing plasmid that are then transfected with siC or siTYMS and cultured in medium containing 5-FU, DMSO or untreated control (right panel). **D** MTT assay (mean ± s.d., *N* = 3) in HCT116 doxycycline inducible NAA40-KD2 cells transduced with the indicated plasmids and grown in DMSO, 5-FU or Dox+5-FU. **E** Mean tumour volume from 5-FU or saline treated HCT116 xenografts stably overexpressing (o/e) NAA40(WT)-V5 or carrying an empty vector control. The tumour volume is shown as mean ± s.e. **F** Tumour weight and **G** representative images of excised empty vector control or o/e NAA40 (WT)-V5 xenograft tumours from mice receiving 5-FU or saline. **H** Correlation between the expression of *NAA40* and *TYMS* in 380 primary colorectal cancer tissues extracted from the TCGA database. The orange line demonstrates the regression slope. Statistical analysis was performed using Pearson’s rank correlation coefficient (r). **I** Relative *NAA40* mRNA levels from two independent datasets extracted from GEO omnibus of patients responding to FOLFOX treatment (“R”) and patients not responding (“NR”). The p values were calculated using Fisher method (*p < 0.05). All other statistical analyses in the figure were performed using unpaired two-tailed Student’s *t* test (ns = no significance, *p < 0.05, **p < 0.01, ***p < 0.001, ****p < 0.0001).

Apart from its role in methyl group biogenesis to support methylation reactions, one-carbon metabolism is also essential for nucleotide metabolism [11]. Specifically, TYMS is a key 1 C metabolic enzyme converting deoxyuridine monophosphate (dUMP) to deoxythymidine monophosphate (dTMP) by competing with the MTHFR enzyme for the one-carbon unit methylenetetrahydrofolate (me-THF) (Fig. 1D). The antimetabolite drug 5-Fluorouracil (5-FU), which is used in first line chemotherapy of CRC [22, 23], targets TYMS by antagonizing dUMP binding on its catalytic domain inhibiting dTMP synthesis (Fig. 5A). Although *TYMS* expression levels have been shown to predict response of malignant cells to 5-FU therapy [24, 25], the mechanism underlying TYMS regulation remains largely unexplored.

Given that we identified *TYMS* to be significantly downregulated upon NAA40-knockdown (Fig. 1A, C) and the fact that Cancer Dependency Map (DepMap) data analysis shows significant positive correlation (r = 0.43, *p* < 0.001) between *NAA40* and *TYMS* expression in 70 different CRC cell lines (Supplementary Fig. S6A), we speculated that NAA40 could be a novel regulator of TYMS affecting the response of CRC cells to 5-FU. To test this hypothesis, we initially sought to verify that *TYMS* expression is indeed responsive to NAA40 levels. Conversely to the observed downregulation of *TYMS* in NAA40-depleted cells (Fig. 1C), we found that *TYMS* levels are increased in cells overexpressing a wild-type but not a catalytically inactive NAA40 relative to empty vector control cells (Fig. 5B), further supporting the regulatory link between NAA40 and TYMS expression.

In light of the above results, we then investigated the effects of NAA40-mediated *TYMS* regulation on the response of CRC cells to 5-FU. We initially examined the response of HCT116 cells overexpressing NAA40 to 5-FU by monitoring cell viability. Interestingly, the viability of cells overexpressing wild-type NAA40-V5 was significantly less affected by the 5-FU treatment compared to non-overexpressing cells carrying an empty vector control (Fig. 5C, right plot). However, TYMS knockdown by transient siRNA (Fig. 5C, left plot) reversed the acquired 5-FU resistance of NAA40(WT)-V5 overexpressing cells, indicating that *TYMS* upregulation is required for NAA40-mediated 5-FU resistance (Fig. 5C, right plot). Consistent with this result, combinatorial supplementation of doxycycline and 5-FU in cells overexpressing the shRNA-resistant wild-type NAA40 conferred resistance to 5-FU when compared to empty vector control cells (Fig. 5D). Most importantly, cells overexpressing an exogenous shRNA-resistant catalytically inactive NAA40 remained sensitive to 5-FU upon doxycycline treatment (Fig. 5D). This result indicates that NAA40 histone acetyltransferase activity is driving 5-FU resistance, in agreement with the fact that this activity is implicated in *TYMS* expression (Fig. 5B).

Following the above in vitro findings, we next wanted to determine if NAA40 controls the response of CRC cells to 5-FU in an in vivo model. Therefore, we inoculated HCT116 cells either stably overexpressing wild-type NAA40-V5 or transfected with the empty vector control into nude immunodeficient mice, followed by treatment with 5-FU or vehicle (saline) control through intraperitoneal injection. As expected, the growth of empty vector control tumours was significantly reduced when treated with 5-FU compared to saline control. In contrast, xenograft tumours overexpressing NAA40-V5 developed with similar growth rate in mice receiving either 5-FU or saline control (Fig. 5E), demonstrating that NAA40 confers 5-FU resistance to CRC tumours similarly to the aforementioned cell-based assays. In addition to the delayed tumour growth, NAA40 overexpressing explants in mice treated with 5-FU showed increased tumour weight and size compared to the empty vector tumours in the equivalent treatment group (Fig. 5F, G). It is worth noting that, in agreement with our in vitro studies (Fig. 5C), the tumour volume of empty vector grafts was vastly similar to that of NAA40 overexpressing tumours receiving saline control, suggesting that NAA40 overexpression alone is not sufficient to promote additional growth advantage to CRC cells (Fig. 5E). Overall, the data from mouse models combined with our in vitro findings indicate that NAA40 promotes 5-FU drug resistance by controlling one-carbon metabolism and particularly *TYMS* expression.

Finally, we explored the clinical relevance of NAA40 regulating *TYMS* expression and 5-FU drug response. In accordance with the existing literature, *NAA40* and *TYMS* were found to be elevated in colorectal cancer patients as opposed to normal colon tissues [5, 24] (Supplementary Fig. S6B). To this end, we performed *in silico* analysis of 380 primary colorectal tumour tissues retrieved from The Cancer Genome Atlas (TCGA) database and found that *NAA40* transcript levels in these patients are positively correlated (r = 0.30, *p* < 0.001) with the expression of *TYMS* (Fig. 5H), consistent with the above-described regulatory link within CRC cells. Moreover, analysis of microarray data derived from two independent cohorts showed that CRC patients defined as non-responders to 5-FU based chemotherapy (FOLFOX) display higher *NAA40* expression levels relative to those who responded well to 5-FU therapy (Fig. 5I), suggesting that NAA40 upregulation in human cancers is associated with resistance to antimetabolite chemotherapy. Altogether, these results indicate that NAA40-mediated regulation of *TYMS* expression affects the response to 5-FU based chemotherapy, providing insight on a new molecular link implicated in CRC drug resistance.

### The NAA40 antagonizing mark H2A/H4S1ph is enriched at the nuclear lamina and mediates *TYMS* repression

**Fig. 6:**
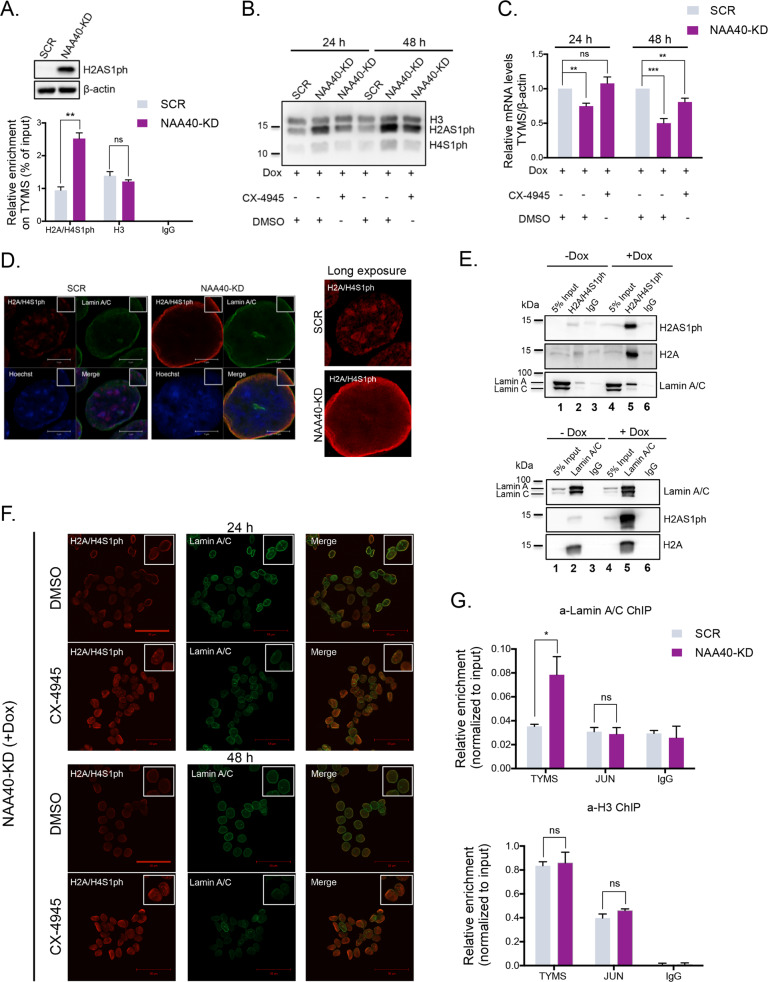
The NAA40 antagonizing mark H2AS1ph is localized at the nuclear periphery and silences *TYMS* expression. **A** Western blot analysis of cell extracts derived from dox-treated SCR or NAA40-KD2 HCT116 cell lines using antibodies against H2A/H4S1ph and β-actin (top panel). ChIP-qPCR (mean ± s.d., *N* = 3) in dox-treated SCR and NAA40-KD2 cells monitoring the enrichment of H2AS1ph, H3 and IgG on *TYMS* gene relative to input (bottom panel). **B** Western blot analysis (*N* = 3) and **C** qRT-PCR analysis (mean ± s.d., *N* = 3) in dox-treated SCR and NAA40-KD2 HCT116 cells cultured with CX-4945 or DMSO for 24 h and 48 h. **D** Representative super-resolution confocal images of H2A/H4S1ph (red) and Lamin A/C (green) in SCR and NAA40-KD2 cells treated with doxycycline for 72 h (left panel). Scale bar, 5 μm. Higher exposure confocal images of H2A/H4S1ph in doxycycline-treated SCR and NAA40-KD2 cells (right panel). **E** Immunoprecipitation of H2A/H4S1ph (top panel) or Lamin A/C (bottom panel) followed by immunoblot analysis (*N* = 3) probed with the indicated antibodies in inducible NAA40-KD2 cells treated or not with doxycycline for 72 h. **F** Confocal IF images of H2A/H4S1ph (red) and Lamin A/C (green) in HCT116 inducible NAA40-KD2 cells following treatment with doxycycline and CX-4945 or DMSO control for 24 h or 48 h. Scale bar, 50 μm. **G** ChIP-qPCR analysis on *TYMS* and *JUN* genomic location using antibodies against Lamin A/C, H3 or IgG control in SCR and NAA40-KD2 treated with dox for 72 h. The enrichment for each antibody was normalized to input. All statistical analyses were performed using unpaired two-tailed Student’s t-test (ns=no significance, *p < 0.05, **p < 0.01, ***p < 0.001).

The above results indicate that *TYMS* expression strongly correlates with NAA40 activity and drug response. Thus, we next looked into possible mechanisms through which NAA40 could mediate transcriptional regulation of *TYMS*. Because NAA40 affects *TYMS* activation through its acetyltransferase activity (Fig. 5B) that is known so far to selectively target histones [21], we turned our focus on the NAA40 antagonizing mark H2A/H4S1ph which has been previously implicated in transcriptional repression [6, 26]. In addition to the robust increase in the bulk levels of H2AS1ph shown by western blot, ChIP analysis showed higher occupancy of H2A/H4S1ph on the *TYMS* gene in NAA40-knockdown cells (2.5-fold increase) compared to SCR control cells (Fig. 6A), which is consistent with *TYMS* downregulation under these conditions (Fig. 1A, C). To further support the connection between H2A/H4S1ph and *TYMS* repression, we examined its expression after exposing doxycycline-treated SCR and NAA40-KD cells to CX-4945 (Silmitasertib), a selective inhibitor of kinase CK2α which mediates H2A/H4S1ph. Importantly, we found that treatment of NAA40-depleted cells with CX-4945 reduced the levels of both H2A and H4 serine 1 phosphorylation and this was accompanied by restoration of *TYMS* expression (Fig. 6B,C).

To define how H2A/H4S1ph could mediate transcriptional silencing upon NAA40 knockdown, we next examined the sub-nuclear localization of this repressive histone mark by super-resolution confocal microscopy. Remarkably, upon NAA40 depletion H2A/H4S1ph re-localizes from the nuclear interior to a prominent ring-like distribution around the inner nuclear membrane of the nuclear envelope where it colocalizes with Lamin A/C (Fig. 6D, Supplementary Fig. S7A, B), a compartment typically associated with transcriptionally repressive heterochromatin [27]. Specifically, the distribution of H2A/H4S1ph at the nuclear periphery increases from 20% in SCR cells to 94% in NAA40-KD cells. In addition, introduction of an shRNA resistant wild-type NAA40 protein into NAA40-deficient cells markedly restored H2A/H4S1ph localization at the nuclear interior (Supplementary Fig. S8). To corroborate the association between H2A/H4S1ph and Lamin A/C, we also performed co-immunoprecipitation (co-IP) experiments through which we detected enhanced interaction between Lamin A/C and H2AS1ph upon doxycycline-induced depletion of NAA40 (Fig. 6E, compare lane 2 with 5).

Consistent with *TYMS* de-repression seen upon CX-4945 treatment (Fig. 6C), the ring-like enrichment of H2A/H4S1ph at the nuclear periphery in NAA40-depleted cells was suppressed after inhibition of CK2α (30% of cells) but not in control DMSO-treated cells (90% of cells) (Fig. 6F). These results suggest that since H2A/H4S1ph becomes enriched at the nuclear periphery upon NAA40 knockdown then *TYMS* might also become associated with the nuclear lamina. Hence, we next examined the occupancy of Lamin A/C at the *TYMS* genomic locus in cells expressing or lacking NAA40. ChIP analysis revealed significantly increased occupancy of Lamin A/C at the *TYMS* gene upon loss of NAA40 but not in SCR control cells, whereas no significant binding was detected at the control *JUN* promoter (Fig. 6G) [28]. Taken together, these data highlight a role for NAA40 in controlling the abundance and localization of its antagonizing H2A/H4S1ph mark at the heterochromatin-associated nuclear lamina thus preventing *TYMS* transcriptional silencing.

## Discussion

Metabolic dysfunction is one of the major hallmarks of cancer and emerging studies are highlighting that epigenetic mechanisms could prompt this dysregulation [29–31]. However, the contribution of this cross-regulation in therapeutic resistance is underexplored. Here, we have focused our studies on deciphering the molecular role of NAA40 in colorectal cancer in which it was previously implicated [5] and we have established a new function for this enzyme in bridging epigenetic regulation and metabolism that is exploited by cancer cells to counteract anti-metabolite drug therapy. It is important to note that the principle of resistance established here might have broader implications in other cancers since NAA40 is upregulated in various types of tumours [4], many of which are routinely treated by chemotherapy regimens encompassing anti-metabolite agents and may develop non-genetically induced chemoresistance [32, 33].

**Fig. 7:**
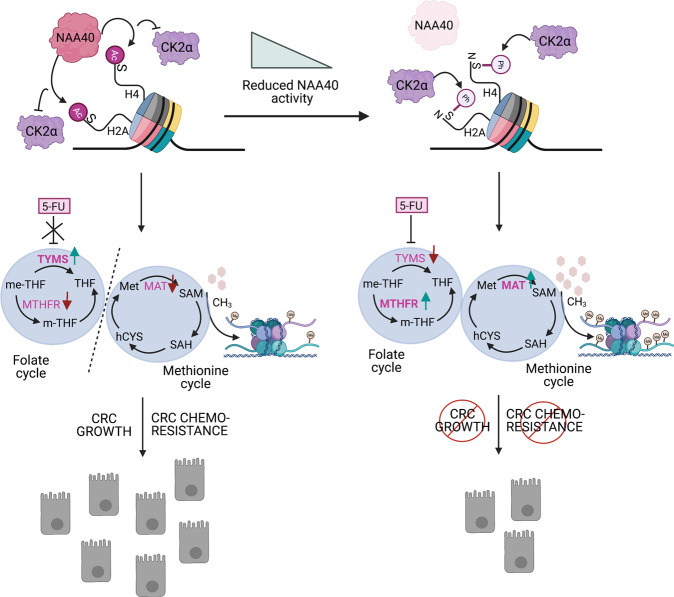
Model for the NAA40-mediated effects on cancer cell growth and chemoresistance through regulation of one-carbon metabolism. High NAA40 activity towards histones H2A and H4 in colorectal cancer (CRC) cells antagonizes CK2α-mediated H2A/H4S1ph inducing *TYMS* expression and promoting resistance to 5-FU anti-metabolite agent. At the same time, NAA40 upregulation decreases *MTHFR* and *MAT1A* levels resulting in low SAM concentrations, reduced global histone methylation and contributing to CRC cell growth. Conversely, loss of NAA40 activity results in the deposition of H2A/H4S1ph at the nuclear periphery, downregulation of *TYMS* and increased sensitivity to 5-FU, whereas concomitant upregulation of *MTHFR* and *MAT1A* lead to high SAM abundance, increased bulk histone methylation levels and reduced CRC cell growth. Image created with BioRender.com.

During this study, we combined transcriptomics and metabolomics analysis to reveal that NAA40 modulates two inter-connected parts of the one-carbon metabolic network which impact on one side methylation reactions and on the other side nucleotide biosynthesis. Specifically, among the deregulated genes found in our RNA-seq analysis we identified a set of genes encoding metabolic enzymes implicated within the methionine cycle. Subsequently, LC/MS analysis demonstrated that NAA40 depletion profoundly affects the abundance of critical intermediary methionine and one carbon cycle metabolites, such as methionine, SAM and UMP, which are intimately connected to the deregulated metabolic enzymes. Accordingly, 1 C metabolic rewiring in response to loss of NAA40 or its histone acetyltransferase activity induces global histone methylation which attenuates CRC cell growth. Importantly this rewiring can be reverted if the methionine and folate cycles are uncoupled by preventing *MTHFR* expression. This finding strongly suggests that NAA40 upregulation in colorectal cancer cells [5] serves to dampen SAM production and associated chromatin methylation in order to sustain malignant properties (Fig. 7). In particular, elevated SAM abundance upon NAA40 knockdown is associated with a pronounced increase in various histone methylation marks since we have demonstrated enhancement in the total levels of H3K4me3, H3K36me3, H3K79me2, H3K9me3 and H3K27me3, whereas H3K79me3 remained unaffected. In line with this, it was previously reported that alterations in SAM abundance mediated either by dietary interventions or disruption of relevant metabolic enzymes, such as Nicotinamide N-methyltransferase (NNMT), impacts several methylated histone residues but the effects are not widespread [16, 17]. It was suggested that this diverse response to SAM abundance could stem from the different affinities (K_m_ values) of particular methyltransferase enzymes for SAM or the diverse turnover rates of individual histone methylation marks [9]. Generally, H3K4me3, H3K36me3, H3K9me3, H3K27me3 and H3K79me2 have been shown to be highly sensitive to changes in SAM levels, whereas H3K79me3 and H3R17me2a were found to be less responsive to such metabolic alterations [16, 17]. Moreover, some of the affected methylated sites have been previously ascribed roles as methyl sinks in order to maintain SAM homeostasis [34].

At the expense of chromatin methylation, one-carbon groups could also be consumed by TYMS for the production of the nucleotide dTMP supporting DNA synthesis and drug resistance. In the current study we show that high levels of NAA40 are tightly associated with lower sensitivity of CRC cells to 5-FU antimetabolite drug in cell-based assays, xenograft tumours and human primary cancer tissues. At the molecular level, we show that NAA40-mediated resistance of CRC cells to 5-FU is dependent on the transcriptional regulation of the one-carbon metabolic gene *TYMS* whose encoded enzyme is directly targeted by 5-FU (Fig. 7). Importantly, 5-FU is the frontline regimen for patients with colorectal cancer. Although considerable progress has been made in the diagnosis and treatment of this malignancy, CRC remains a major cause of cancer related mortality in both genders mainly as a result of developed resistance to 5-FU-based chemotherapy [22, 23]. Therefore, there is an urgent need to identify biomarkers that would predict poor drug response and thus eliminate disease recurrence. In addition, insights for new combinatorial therapies are needed in order to improve the efficacy of current 5-FU based chemotherapies. For instance, a recent study has shown that modulating one-carbon metabolism by methionine restriction can synergize with 5-FU to inhibit CRC cell growth [35]. This synergistic effect possibly lies in the fact that during methionine deprivation there is an increased flux of carbon units into the methionine cycle leading to histone methylation reprogramming which antagonizes dTMP synthesis by TYMS thus rendering cells more responsive to 5-FU [35, 36]. It would be interesting to determine in the future if NAA40 can act as a molecular sensor coupling nutrient availability to one-carbon metabolism. Nonetheless, our data argue that targeting NAA40 could be part of such combinatorial therapies and this prospect is further supported by the fact that this enzyme can be specifically inactivated by potent small-molecule inhibitors that have been recently discovered [37].

Our findings unveil the mechanism through which NAA40 regulates *TYMS* expression. Specifically, we provide evidence that *TYMS* silencing in NAA40-deficient cells is controlled by CK2α-mediated H2A/H4S1ph which has been previously reported to negatively crosstalk with NAA40-mediated histone N-terminal acetylation and inhibit transcription [6, 26]. Nevertheless, we show for the first time to our knowledge that the NAA40 antagonizing mark H2A/H4S1ph is strikingly redistributed from the interior of the nucleus to the nuclear periphery where it interacts with Lamin A/C. Co-enrichment of H2A/H4S1ph and Lamin A/C on the genomic locus of the one-carbon metabolic gene *TYMS* upon NAA40 depletion could mediate *TYMS* repression and thus reduced resistance against 5-FU. Since anchoring of chromatin to the nuclear lamina associates with heterochromatin compartments and transcriptional silencing [38, 39], our findings provide new insight on the repressive nature of H2A/H4S1ph which could serve as a critical factor for heterochromatin organization at the nuclear periphery driving gene inactivation and halting cancer-associated phenotypes. This implication of H2A/H4S1ph could be of particular importance in the future since it was recently shown that in tumour tissues substitution of serine (S) to cysteine (C) at position 1 is the most frequently occurring mutation on histone H2A and the second most frequent mutation on histone H4, further signifying the value of S1 modifications in carcinogenesis [40]. Moreover, subsequent studies are needed to explore whether re-localization of H2A/H4S1ph at the nuclear periphery in the absence of NAA40 influences the expression of other genes, since the nuclear lamina serves as a docking site for several genes [41, 42].

To conclude, our data show that NAA40 controls key metabolic genes to promote nucleotide synthesis and resistance to antimetabolite therapy. Hence, our results strongly favor the idea that NAA40 is a critical mediator at the interface between epigenetics and metabolism by linking one-carbon cycle to drug response and signify its potential as a novel predictive factor and therapeutic target in colorectal cancer.

## Materials and methods

### Cell culture

The HCT116 cell line was kindly provided by Dr. Pantelis Hatzis (Biomedical Sciences Research Center ‘Alexander Fleming’) and the HT-29 (catalogue no. HTB-38), SW480 (catalogue no. CCL-228) and SW620 (catalogue no. CCL-227) cell lines were purchased from ATCC. All CRC cell lines were cultured in McCoy’s 5a medium (Gibco, Invitrogen) supplemented with 10% fetal bovine serum (Gibco, Invitrogen) and 1% penicillin/streptomycin (Gibco, Invitrogen). The human embryonic kidney HEK-293 T (catalogue no. CRL-3216) cell line was purchased from ATCC and was cultured in DMEM medium (Gibco, Invitrogen) supplemented with 10% fetal bovine serum and 1% antibiotic (penicillin/streptomycin). Cells were grown in a humidified atmosphere at 37 °C containing 5% CO_2_ and were routinely tested for mycoplasma contamination. All cell lines were used to construct dox-inducible shRNA-knockdown lines for NAA40 or Scramble (SCR) control as previously described (Demetriadou et al). For CK2α inhibition cells were treated with 7 μM or 5 μM of CX-4945/Silmitasertib (HY-50855, MCE) for 24 h and 48 h, respectively.

### Lentiviral overexpression of wild-type and mutant NAA40 in CRC cells

The pLenti/p53-V5_wt plasmid (Addgene, #22945) containing a C-terminal V5 tag was used in which p53 was replaced with the full-length human NAA40 cDNA subcloned from pOTB7 vector between BamHI and XhoI restriction sites. As a control, pLenti/V5-empty vector (Empty vector) was used that is not encoding for anything between the two restriction sites. To generate the shRNA-resistant constructs, six silent mutations were introduced by site-directed mutagenesis using the Pfu Turbo DNA polymerase (Agilent Technologies) in the region of NAA40 cDNA that is targeted by NAA40-KD2 shRNA (5’-GAAAGTGATGCTGACGGTGTT-3’ where substituted nucleotides are underlined) hence constructing the shRNA-resistant wild-type pLenti/NAA40-V5rescue plasmid (Resistant NAA40(WT)-V5). The synonymous mutations have been introduced sequentially using three different sets of primers (Supplementary Table S1). Catalytically dead Resistant NAA40(E139Q)-V5 plasmid was generated by site-directed mutagenesis of the pLenti/NAA40-V5rescue vector. The primers used for the site-directed mutagenesis were purchased from Integrated DNA Technologies (IDT) (Supplementary Table S1). For lentiviral packaging, each of the recombinant vectors was co-transfected with the psPAX2 lentivirus packaging vector and the PMD2G lentivirus envelope plasmid in HEK-293T cells by using X-tremeGENE 9 DNA transfection reagent (Roche) according to manufacturer’s instructions. Upon 48 h of transfection the virus containing supernatant was collected and used to stably infect doxycycline inducible HCT116/NAA40-KD cells in the presence of 10 μg/ml polybrene. The pool of efficiently transduced cells was selected in complete McCoy’s 5a medium containing 20 μg/ml BlasticidinS-HCL (A1113903, Thermo Fisher Scientific) for 4 d. For the shRNA induction, cells were treated with doxycycline hyclate (Sigma–Aldrich) at an assay dependent concentration and time period.

### Transient RNA interference

HCT116 cell lines were seeded in antibiotic-free medium and grown to 40% confluence at the time of transfection. Subsequently, the cells were transiently transfected with 20 nM of siMTHFR (4392420, s9036, Ambion) or 10 nM of siTYMS (4392420, s14538, Ambion) or the negative control (4390843, Ambion) for 72 h using Lipofectamin RNAiMAX (Invitrogen) according to manufacturer’s instructions. MTHFR knockdown was preformed in HCT116/SCR and HCT116/NAA40-KD stable cells in the presence or absence of 1 μg/ml doxycycline. For TYMS silencing experiments, the transiently transfected HCT116 Empty vector or o/e NAA40(WT)-V5 stable cells were also treated with 5 μM of 5-FU (F 6627, Sigma) or DMSO control.

### RNA extraction and quantitative Real Time PCR (qRT-PCR)

Total RNA was extracted using the RNeasy Mini kit (Qiagen) according to the manufacturer’s instructions and was then treated with DNAse using the TURBO DNAse kit (Ambion). An amount of 0.5 μg total RNA was then reverse transcribed to complementary DNA using the PrimeScript RT reagent kit (Takara) with random primers. qRT-PCR was carried out using KAPA SYBR Green (SYBR Green Fast qPCR Master Mix) and the Biorad CFX96 Real-Time System. Expression data were normalized to the mRNA levels of the β-actin housekeeping gene and calculated using the 2^−ΔΔCt^ method. Primer sequences were obtained from IDT (Supplementary Table S2).

### RNA-sequencing and bioinformatics analysis

Total RNA was isolated from the HCT116/SCR and HCT116/NAA40-KD engineered cells in the presence or absence of 1 μg/ml dox for 96 h using the RNeasy mini kit (Qiagen) according to manufacturer’s instructions. Four independent RNA samples were prepared from each of the four conditions: SCR (−dox), SCR (+dox), NAA40-KD (−dox) and NAA40-KD (+dox). Efficient NAA40 knockdown was evaluated through qRT-PCR using specific primers against NAA40 and β-actin (Table S2). Total RNA was isolated from the HCT116/SCR and HCT116/NAA40-KD engineered cells in the presence or absence of 1 μg/ml dox for 96 h using the RNeasy mini kit (Qiagen) according to manufacturer’s instructions. Four independent RNA samples were prepared from each of the four conditions: SCR (−dox), SCR (+dox), NAA40-KD (−dox) and NAA40-KD (+dox). Efficient NAA40 knockdown was evaluated through qRT-PCR using specific primers against NAA40 and β-actin (Supplementary Table 2). Sequencing libraries were prepared using the NEBNext stranded RNA library prep kit according to the manufacturer’s protocol. Sequenced reads were aligned to the mm10 genome via STAR (v 2.4.1b) [43]. Gene counts were calculated using featureCounts of the Rsubread package (R/Bioconductor). Only reads with counts per million >1 were kept for subsequent analysis. Counts were normalized using the internal TMM normalization in edgeR [44] and differential expression was performed using the limma package [45]. Significant genes with an absolute logFC > 1 and adjusted *P* < 0.05 were considered differentially expressed. For examination of NAA40 expression through the cell cycle we examined publicly available expression data from synchronized primary human fibroblasts (GSE104616). Data were obtained from GEO omnibus for the single NAA40 probe (7940824) contained within the microarray platform used (GPL11532) and no further processing was performed.

### Metabolite extractions

HCT116 SCR and NAA40-KD cells were seeded at a density of 1.5 × 10^5^ cells/ml and treated with 4 μg/ml doxycycline for 24 h. Metabolites were extracted from cells using a modified method of Folch and colleagues [46]. Briefly, 5 × 10^6^ cells were homogenized in chloroform/methanol (2:1, v/v, 750 μL). Samples were sonicated for 15 min and deionized water was added (300 μL). The organic and aqueous phases were separated following centrifugation (13,000 x g for 20 min). The resulting organic and aqueous phases were dried under a stream of nitrogen gas and a vacuum centrifuge, respectively.

### Metabolomic analysis

Aqueous extracts were reconstituted in acetonitrile 10 mM ammonium carbonate (7:3, v/v, 50 μL) containing an internal standard mix (AMP 13C10, 15N5; ATP 13C10, 15N5; Glutamate U13C, U15N; Leucine-d10, Phenylalanine-d5, Proline U13C, U15N; and Valine-d8). Samples were injected onto a Vanquish UHPLC attached to a TSQ Quantiva triple quadrupole mass spectrometer (Thermo Scientific) with a heated ESI source.

For the normal phase analysis, metabolites were separated with a BEH-amide (150 ×2.1 mm 1.7 μm) column at 30 °C. The mobile phase consisted of: (A) 0.1% of ammonium carbonate and (B) acetonitrile and was pumped at a flow rate of 0.6 mL/min. The gradient was programmed as follows: 80% of B for 1.50 min followed by a linear decrease from 80% to 40% of B for 3.5 min and finally returned to initial conditions.

For reverse phase analysis, samples were dried and reconstituted in 10 mM ammonium acetate solution and analyzed with an ACE C18 PFP (150 × 2.1 mm 5 µm) column at 30 °C. The mobile phase consisted of: (A) 0.1% formic acid in water and (B) 0.1% formic acid in acetonitrile, pumped at 0.5 mL/min. The gradient was programmed as follows: 0% of B for 1.60 min followed by a linear increase from 0% to 30% of B for 4 min and to 90% by 4.5 min, held for 1 min and then returned back to initial conditions.

The mass spectrometer was operated in SRM mode in both positive and negative ion mode; collision energies and RF lens voltages were generated for each species using the TSQ Quantiva optimization function. Xcalibur Software (Thermo Scientific) was used to identify peaks, process mass spectra and normalize data to the closest-eluting internal standard. All variables were log transformed and subjected to Pathway analysis and metabolite set enrichment analysis of significant metabolites in Metaboanalyst 4.0 (www.metaboanalyst.ca).

### Protein extraction

Protein extracts were isolated using Lysis Buffer (50 mΜ Tris-HCL pH 8, 3 mM EDTA, 100 mM NaCL, 1% Triton-X-100, 10% glycerol, 0.5 mM PMSF and 1X protease inhibitor cocktail) and total protein concentration was quantified by Bradford assay (BioRad). For efficient NAA40 detection, whole cell extracts were resuspended in a tenfold volume of Laemmli sample buffer (50 mM Tris-HCL pH 6.8, 2% SDS, 10% glycerol, 1% β-mercaptoethanol, 12.5 mM EDTA and 0.02% bromophenol blue) and alternatively boiled and chilled three times to disrupt cell membranes. For histone acid extraction, cells were lysed in hypotonic lysis buffer (10 mM Tris-HCL pH 8, 1 mM KCL, 1,5 mM MgCl_2_, 0,1% Triton X-100 and 1X protease inhibitor cocktail) and incubated for 30 min with constant agitation at 4 °C. Isolated nuclei were then washed once in hypotonic lysis buffer and after centrifugation at 6500 g for 10 min, were resuspended in 0,2 M HCL (4 × 10^7^ nuclei per ml) and incubated overnight with constant rotation at 4 °C. Histones were isolated by centrifugation at 6500 g for 10 min and the pH was neutralized with 2 M NaOH at 1/10 of the volume of the supernatant.

### Immunoblotting

Twenty-five micrograms of protein extract, six micrograms of histone extracts or 10% of the laemmli-extracted samples were separated on SDS-PAGE and then transferred to a nitrocellulose membrane (GE Healthcare). After blocking with 5% TBS-T/BSA for 1 h at RT, the membranes were incubated with the primary antibodies overnight at 4 °C. The primary antibodies that were used in this study are listed in Supplementary Table 3. For secondary antibody a Horseradish peroxide (HRP)-conjugated goat anti-rabbit IgG (Thermo Scientific) was used at a dilution of 1:30000 and an HRP-conjugated goat-anti mouse IgG (P0447, Dako) was used at a dilution of 1:1000. The intensity values were normalized against β-actin and are expressed relative to the SCR control.

### Sub-cellular fractionation

Ten million cells were harvested in 1X PBS and lysed in Buffer A (10 mM HEPES, 10 mM KCL, 1.5 mM MgCl_2_, 0.34 mM sucrose, 10% glycerol, 0.1% Triton X-100 and 1X protease inhibitor cocktail) on ice for 10 min. Following centrifugation at 1300 g for 5 min at 4 °C, the supernatant S1 was centrifuged at maximum speed for 10 min and the supernatant S2 was taken as the cytoplasmic fraction. Pellet P1 was washed in Buffer A (without 0.1% Triton X-100), lysed in Buffer B (3 mM EDTA, 0.2 mM EGTA, 10 mM HEPES and 1X protease inhibitor cocktail) for 30 min on ice and pelleted at 6000 g for 10 min to obtain supernatant S3 that represents the nucleoplasmic fraction. The insoluble chromatin pellet P3 was then washed twice in Buffer B and resuspended in 1X Laemmli sample buffer. For whole cell extract control ten million cells were resuspended in 1X Laemmli sample buffer and alternatively boiled and chilled three times.

### Chromatin immunoprecipitation (ChIP) assay

Doxycycline treated SCR and NAA40-KD HCT116 cells were first fixed in 1% PFA for 10 min and quenched with 125 mM of glycine for 10 min. After the cells were lysed in SDS lysis buffer (1% SDS, 10 mM EDTA, 50 mM Tris-HCL pH 8 and protease inhibitor cocktail), the DNA was sheared by sonication (40 sec ON/40 sec OFF for 6 cycles) in a Bioruptor (Diagenode) to obtain chromatin fragments between 300 and 800 bp. The chromatin was diluted 1:10 in IP buffer (1% Triton X-100, 2 mM EDTA, 50 mM Tris-HCL pH 8, 150 mM NaCl and protease inhibitor cocktail) followed by 1 h pre-clearing in a 1:1 A/G Sepharose beads mix (#17-5280-01 and #17-0618-01, GE Healthcare) at RT. Thirty micrograms of chromatin were incubated with H4/H2AS1ph (ab177309, Abcam; 3 μg), Lamin A/C (sc-7292X, Santa Cruz; 5 μg), H3 (ab1791, Abcam; 2 μg) or IgG (Biogenesis 5180-2104) antibodies for 1 h at 4 °C and subsequently 50% slurry protein A or G beads (blocked in salmon sperm DNA and BSA) were added and incubated overnight at 4 °C. Following washing steps, the immunoprecipitated chromatin was eluted in freshly prepared elution buffer (1% SDS and 0.1 M NaHCO3) and reverse cross-linked using 200 mM NaCl containing 0.5 μg/μl RNase (Roche) at 65 °C for 5 h. The samples were purified using the QIAquick PCR purification kit (QIAGEN) and analyzed with qRT-PCR. The sequence of the primers used in this analysis are listed in Supplementary Table 2.

### Co-immunoprecipitation

Ten million cells were harvested in 1X PBS and lysed in 1 ml ice-cold IP buffer (20 mM Tris-HCL p^H^8, 137 mM NaCl, 1% Triton X-100, 2 mM EDTA and 1X protease inhibitor cocktail) for 30 min with constant mixing at 4 °C. Following centrifugation at 12,000 rpm for 20 min at 4 °C, the soluble supernatant fraction was pre-cleared with Protein A sepharose beads for 1 h at 4 °C. Five percent of the lysate was kept as “Input” to serve as a positive control. Lysates were then mixed with 60 μl of Protein A sepharose beads that were pre-incubated with 4 μg of H4/H2AS1ph (ab177309, Abcam), Lamin A/C (sc-7292 X, Santa Cruz) or IgG (Biogenesis 5180-2104) antibodies for 4 h and blocked in salmon sperm DNA for 40 min. Following overnight incubation with constant agitation at 4 °C, the antibody-beads-protein complexes were centrifuged and washed three times with low salt buffer (10 mM Tris-HCL p^H^7.4, 1 mM EDTA, 1 mM EGTA, 150 mM NaCl, 1% Triton X-100 and 1X protease inhibitor cocktail) and IP samples were eluted in 2X Laemmli buffer at 95 °C for 10 min.

### Immunofluorescence imaging

Cells were fixed in 100% ice-cold methanol at −20 °C for 10 min, washed three times with 1X PBS and further permeabilized in 0.3% Triton X-100 for 10 min. Following blocking in 10% normal goat serum (MP Biomedicals), cells were incubated with the primary antibody in blocking buffer at 4 °C overnight. The following antibodies were used for immunofluorescence: H4/H2AS1ph (ab177309, Abcam; 1:2000), Lamin A/C (ab238303, Abcam; 1:1000), H3K4me3 (ab8580, Abcam; 1 μg/ml) and H3K36me3 (ab9050, Abcam; 1 μg/ml). Next, cells were washed three times with 1X PBS and following incubation with Alexa Fluor 568 goat anti-rabbit (A11011, Thermo Fisher Scientific; 1:1000) and Alexa Fluor 488 goat anti-mouse (A11001, Thermo Fisher Scientific; 1:1000) secondary antibodies diluted in 10% normal goat serum for 1 h at room temperature, nuclei were stained with DAPI (Dako) or Hoechst 33342 (Invitrogen). Samples were imaged on a ZeissAxio Observer.A1 microscope. For confocal and super resolution microscopy imaging was carried out on a ZEISS LSM 900 with Airyscan 2 using Zen blue for acquisition and processing. Airyscan2 images were processed using the default deconvolution settings and histogram stretching, applied when required, was identical between control and treated samples for each channel.

### MTT assay

To assess cell viability, CRC cells were seeded in a 96-well plate at a concentration of 2.5 × 10^4^ cells/ml. At the end of each treatment, 1 mg/ml MTT dye (Invitrogen) was added to each well and then cells were placed at 37 °C for 3 h. The formazan product was solubilized in DMSO and the plate was shaken for 20 min in dark. The absorbance was read at 570 nm by using a Perkin Elmer Wallac Victor 1420-002 Multilabel Counter.

### Cell cycle analysis

Cells were harvested by trypsinization, washed in 1X PBS and fixed in 70% ice-cold ethanol overnight at 4 °C. Fixed cells were pelleted by centrifugation at 2000 rpm for 5 min and resuspended in 1X PBS with 0.2 mg/ml RNase A (12091-021, Invitrogen) and 0.01 mg/ml PI (40017, BIOTIUM). After incubation at 37 °C for 30 min, samples were analyzed using Guava EasyCyte^TM^ flow cytometer and the GuavaSoft analysis software (Millipore, Watford, UK).

### Tumor xenografts in nude mice

The xenograft studies were performed at the animal facility of the Cyprus Institute of Neurology and Genetics under animal project license (CY/EXP/PR.L10/2018) issued and approved by the Cyprus Veterinary Services which is the Cyprus national authority for monitoring animal research for all academic institutions according to the regulations contained in the Cyprus Law N.55 (I)/2013 and the EU Directive 2010/63/EU. A total of 2.5 × 10^6^ HCT116 cells stably transfected with Empty vector or o/e NAA40(WT)-V5 plasmid were suspended in 40 μl of serum-free McCoy’s 5a medium and inoculated subcutaneously in the left flank of 6 week-old male CD1 nude immunodeficient mice. Once the tumors reached an average size of about 50 mm^3^ (day 10) groups were size-matched (*n* = 8) and mice were treated with 5-FU (50 mg/kg every 72 h) or saline vehicle control through intraperitoneal injection. Throughout the experiment, mice were monitored for their overall health condition. Tumor volume was measured twice per week using a digital caliper and calculated using the volume of an ellipsoid and assuming that the third dimension, *z*, is equal to $\sqrt {x\,y}$. Therefore, the volume was given by the equation: $V = \frac{{4\pi }}{3}\frac{{\left( {xyz} \right)}}{8}$. At the end of the experiment, mice were euthanized and tumors were excised, weighted and stored for further processing.

### Meta-analysis of CRC datasets

RNA-seq expression data for colorectal cell lines were obtained from Depmap portal (https://depmap.org/portal/) [47]. The cancer genome atlas (TCGA) data where obtained using the UCSC Xena tool [48]. Pearson’s correlation was then calculated. NAA40 microarray data were extracted from GEO omnibus for two studies of colorectal cancer patients receiving FOLFOX chemotherapy regiment and for which patients were classified into “responders” and “non-responders” (GSE69657 and GSE28702). For both these studies transcriptomics were performed using the same platform (GPL570 [HG‐U133_Plus_2] Affymetrix Human Genome U133 Plus 2.0 Array). The values for the two NAA40 probes in this platform (222369_at and 218734_at) were extracted and averaged without any further normalization. *P* values were calculated using Fisher method.

### Statistical analysis

Statistical analysis was carried out using GraphPad Prism (v.6.01, La Jolla, CA). All presented data are the mean ± s.d. of at least three independent experiments and comparisons between groups were performed using Unpaired Student’s *t* test unless otherwise stated in the figure legend. Differences with ^*^p < 0.05 were considered to be statistically significant.

## Supplementary information

Supplemental Figure legends

Figure S1

Figure S2

Figure S3

Figure S4

Figure S5

Figure S6

Figure S7

Figure S8

Table S1

Table S2

Table S3

## Data Availability

All data needed to evaluate the conclusions in the paper are present in the paper and/or the Supplementary Material. The accession number for the RNA-sequencing data reported here is GSE167474.
