# Supplementary material for: Histone N-terminal acetyltransferase NAA40 links one-carbon metabolism to chemoresistance
Source: Oncogene. 2021 Nov 16;41(4):571–85. doi: 10.1038/s41388-021-02113-9 (PMC8782725; doi:10.1038/s41388-021-02113-9)
Supplement: Supplementary file 1 — Supplemental Figure legends [file 41388_2021_2113_MOESM1_ESM.docx]

**Supplemental figure legends**

**Figure S1. Effects of NAA40 knockdown in cell cycle and metabolite abundance.** (A) Flow cytometry analysis (mean ± s.d., N=2) of HCT116 cells stably transfected with doxycycline-inducible SCR or two independent NAA40-KD (NAA40-KD1 and NAA40-KD2) constructs upon treatment with 1 μg/ml dox for 96 h. (B) Intracellular levels of the indicated metabolites found in one-carbon network (*top panel*) and glycolysis (lower panel) measured by UPLC-MS (mean ± s.e., N=3) in HCT116 SCR and NAA40-KD2 cells treated with doxycycline. Unpaired two-tailed Student’s t-test was used (*p<0.05, **p<0.01).

**Figure S2. NAA40 depletion affects chromatin bound histone methylation.** (A) Western blot analysis (N=3) of acid extracted histones from dox-treated SCR and NAA40-KD2 using antibodies against H2A/H4S1ph, H3, H2A and H4. (B) IF staining (N=2) using antibodies against H3K4me3 and H3K36me3 in dox-treated SCR, NAA40-KD1 and NAA40-KD2 HCT116 cells. Cell nuclei are indicated with DAPI stain. Scale bar, 100 μm. (C) Western blot analysis (N=2) in whole cell (5% Input), nucleoplasmic (Nucl.) and chromatin-bound (Chr.) protein extracts derived from biochemical fractionation of dox-treated SCR or NAA40-KD2 cells.

**Figure S3. NAA40 knockdown reduces cell viability.** MTT assay (mean ± s.d., N=3) of SCR and two independent NAA40-KD (NAA40-KD1 and NAA40-KD2) HCT116 cell lines incubated with dox for the indicated time points. Cell viability is shown as a percentage relative to the corresponding dox untreated cells. Unpaired two-tailed Student’s t-test was used (ns=no significance, *p<0.05, **p<0.01, ***p < 0.001).

**Figure S4. NAA40-KD effects are maintained in the absence of active cell cycle**. (A) Time course of NAA40 expression levels in primary human fibroblasts (NHDF) synchronised by culture in 0.1% Foetal calf serum for 48 hours followed by full media replacement. Two or three replicates were examined at each timepoint, with the symbol representing average expression and the error bars standard deviation. (B) Phase contrast microscopy of doxycycline treated and untreated HCT116 NAA40-KD2 cells cultured in 10%, 0,5% and 0% fetal bovine serum (FBS) for 72h. The images are representative fields from at least three independent replicates. Scale bar, 100 μm. (C) Western blot analysis of NAA40-KD2 cells cultured in 10%, 0,5% or 0% FBS and treated with or without doxycycline for 72h using antibodies against the specified antibodies. (D) qRT-PCR analysis (mean ± s.d., N=3) demonstrating the -Dox/+Dox ratio of *NAA40*, *TYMS* and *MTHFR* mRNA levels normalized to *β-actin* in HCT116 inducible NAA40-KD cells treated with or without doxycycline in the presence of 10%, 0,5% or 0% FBS for 72h.

**Figure S5. NAA40 affects one-carbon metabolism and histone methylation in different CRC cell lines.** (A) Western blot analysis (N=3) of cell extracts derived from dox-treated SCR or NAA40-KD (NAA40-KD1 and NAA40-KD2) HT-29, SW480 and SW620 colorectal cancer cells. (B) qRT-PCR analysis (mean ± s.d., N=3) of *NAA40* and *MTHFR* mRNA levels normalized to β-actin performed in dox-treated HT-29, SW480 and SW620 engineered to express SCR or NAA40-KD2. Student’s t-test was used (**p < 0.01, ***p < 0.001).

**Figure S6. *NAA40* and *TYMS* transcript levels are elevated in CRC cell lines and patient specimens.** (A) Scatter plot demonstrating the correlation between the expression of *NAA40* and *TYMS* in 70 colorectal cancer cell lines. Statistical analysis was performed using Pearson’s rank correlation coefficient (r). The orange line demonstrates the regression slope. (B) Box plots showing the mRNA transcript levels of *NAA40* (left panel) and *TYMS* (right panel) in colorectal (COAD) normal and cancer (CRC) human tissues. Data were obtained from the online Gene Expression Profiling Interactive analysis (GEPIA) database.

**Figure S7. H2A/H4S1ph localizes at the nuclear periphery upon NAA40 knockdown.** (A) Fluorescence intensity profile of H2AS1ph, Lamin A/C and Hoechst signal along the drawn line in doxycycline-treated SCR and NAA40-KD2 cells (confocal images presented in Fig. 6D). Scale bar, 50 μm. (Β) Confocal images of H2A/H4S1ph (red) and Lamin A/C (green) in SCR and NAA40-KD2 HCT116 cells treated with doxycycline for 72 h. Scale bar, 50 μm. Cell nuclei are indicated with Hoechst stain.

**Figure S8. Localization of H2A/H4S1ph at the nuclear periphery is rescued in cells overexpressing wild type NAA40.** Confocal images of H2A/H4S1ph (red) and Lamin A/C (green) in inducible HCT116 NAA40-KD2 cells that stably overexpress the shRNA-Resistant NAA40(WT)-V5 and are treated with doxycycline for 72 h to deplete the endogenous NAA40. Scale bar, 50 μm.
