# Supplementary figures and images for: Histone N-terminal acetyltransferase NAA40 links one-carbon metabolism to chemoresistance
Source: Oncogene. 2021 Nov 16;41(4):571–85. doi: 10.1038/s41388-021-02113-9 (PMC8782725; doi:10.1038/s41388-021-02113-9)

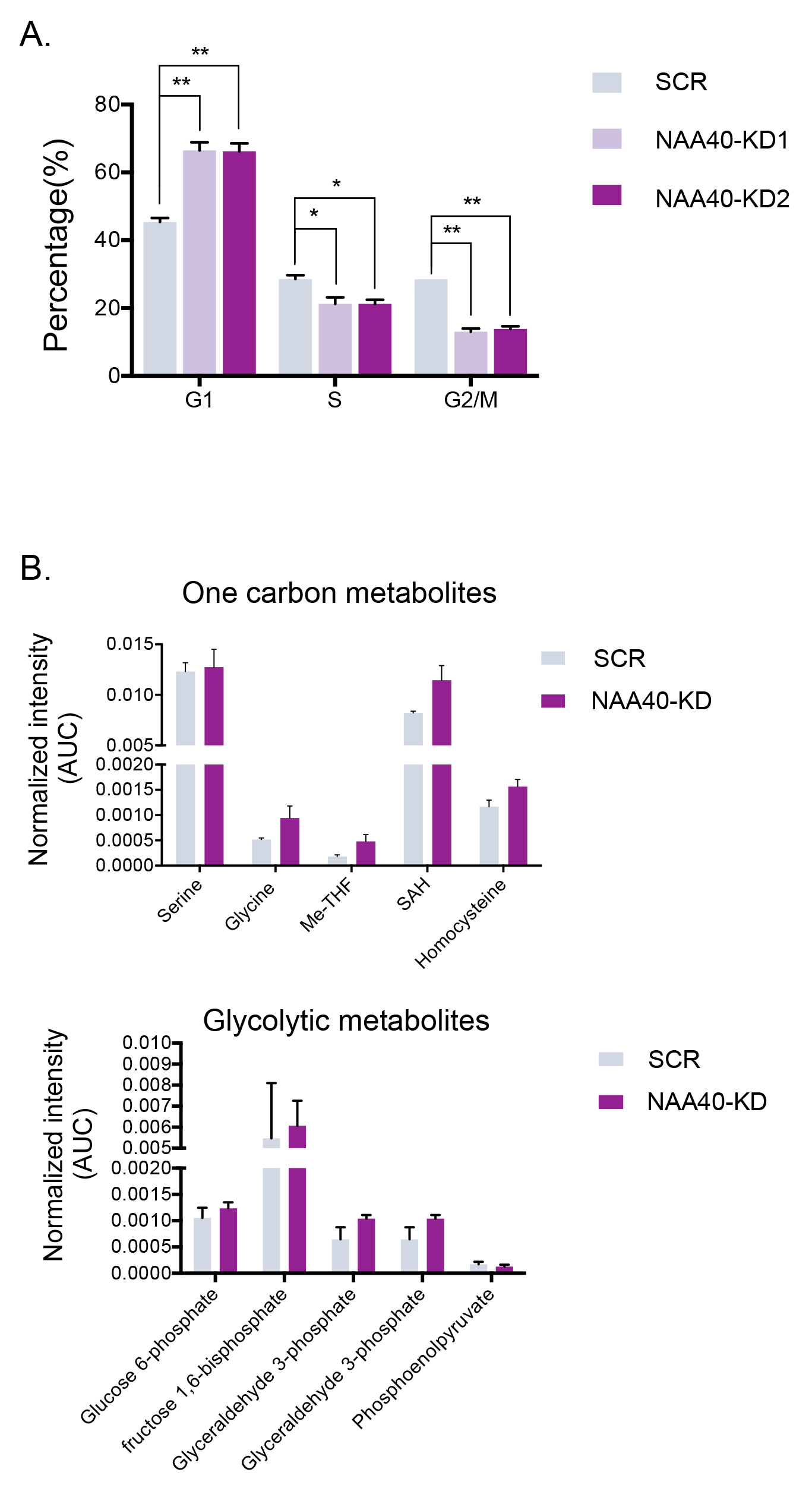

Supplement: Supplementary file 2 — Figure S1 [file 41388_2021_2113_MOESM2_ESM.tif]

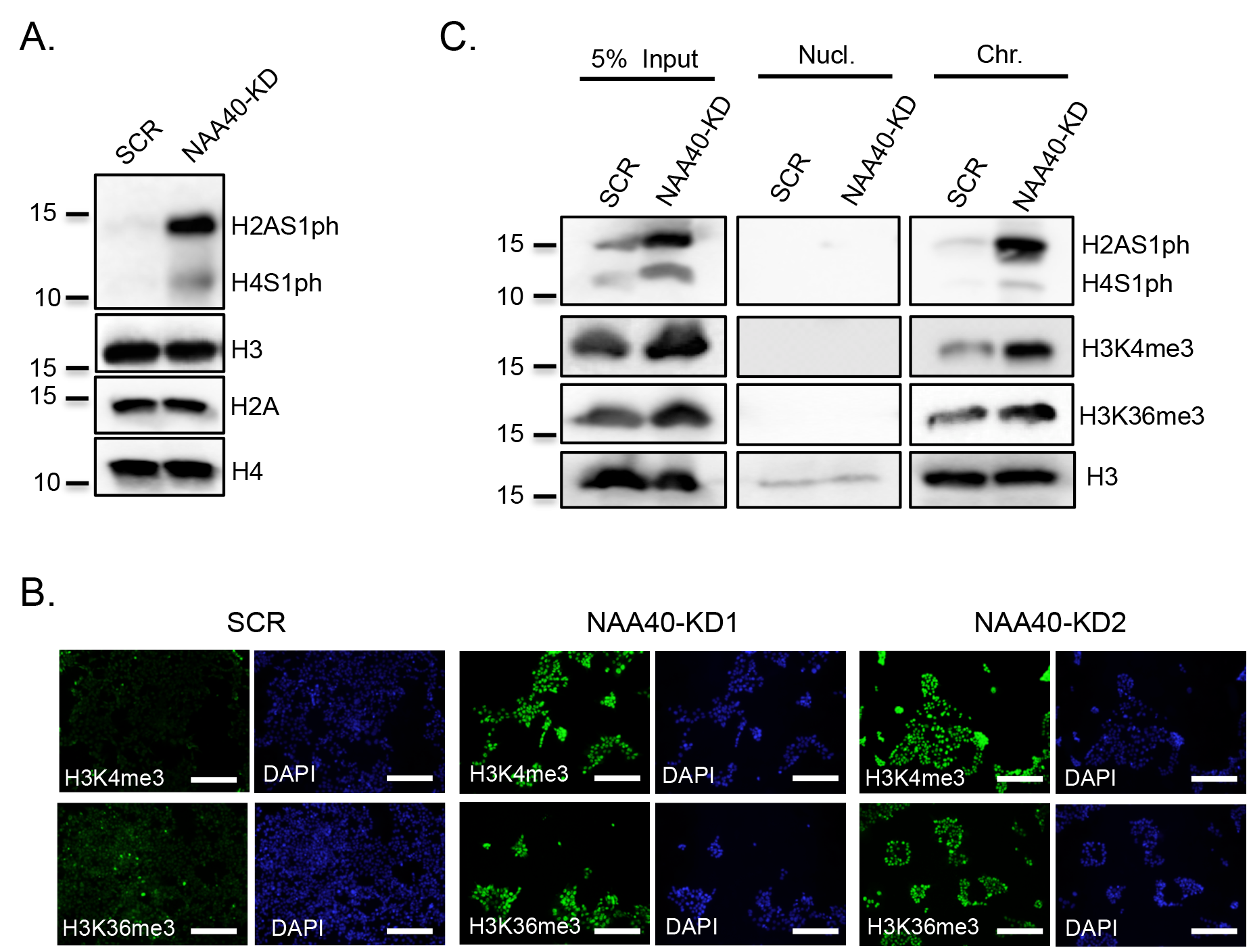

Supplement: Supplementary file 3 — Figure S2 [file 41388_2021_2113_MOESM3_ESM.tif]

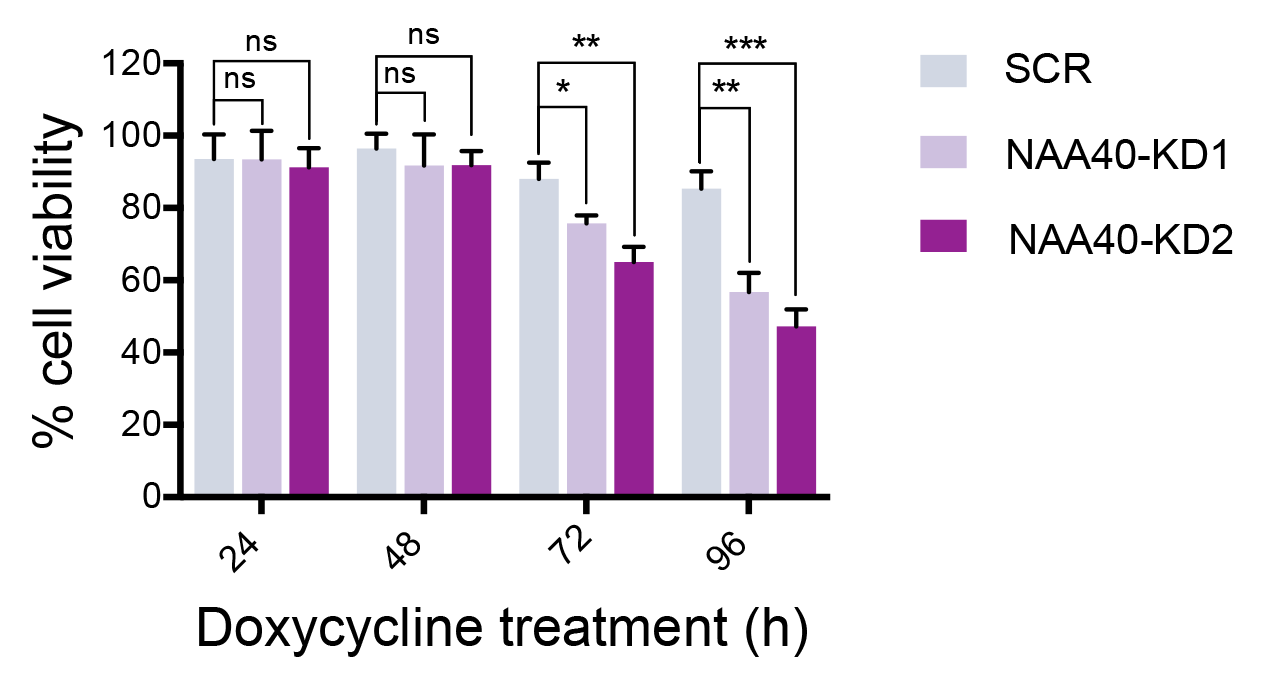

Supplement: Supplementary file 4 — Figure S3 [file 41388_2021_2113_MOESM4_ESM.tif]

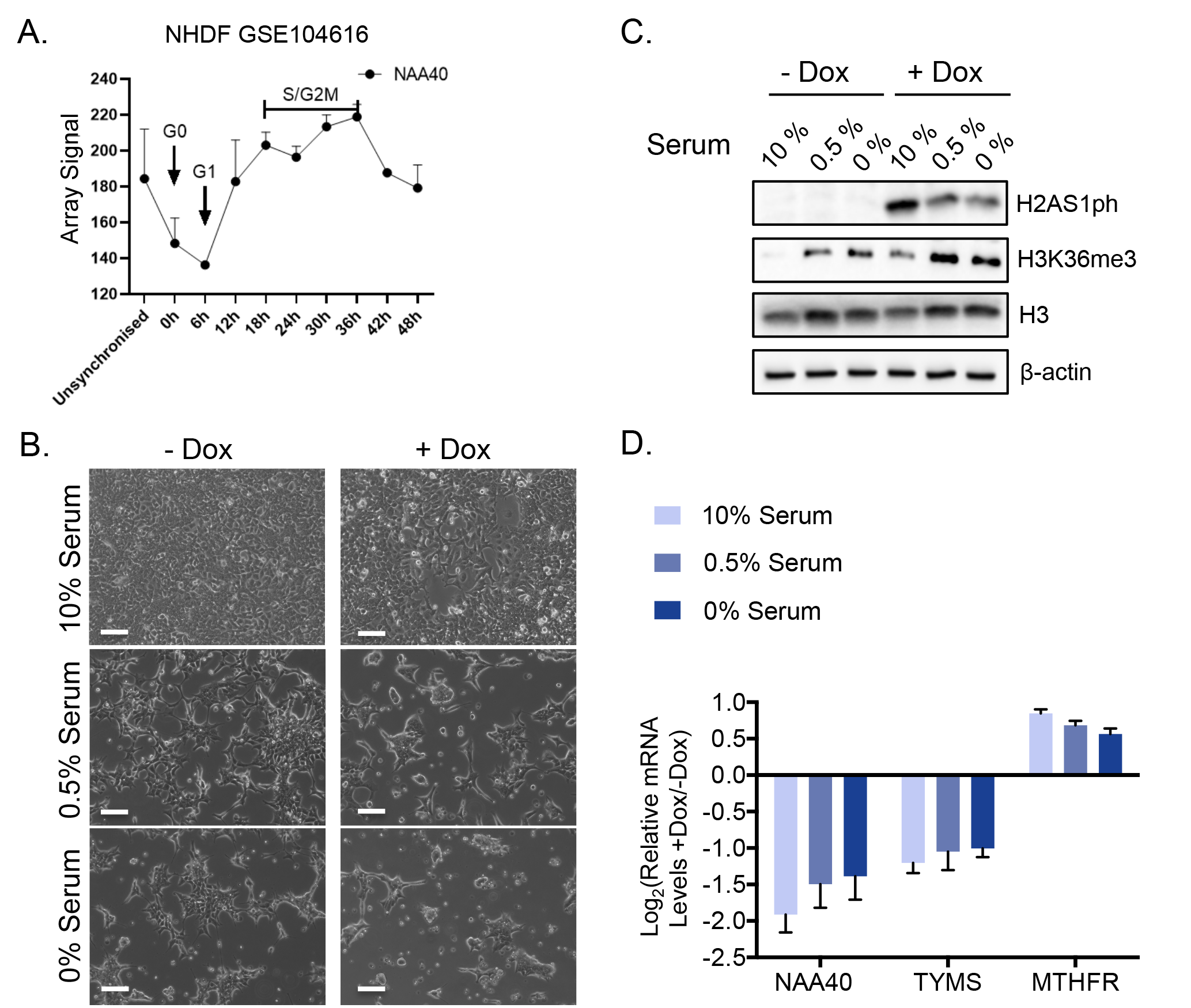

Supplement: Supplementary file 5 — Figure S4 [file 41388_2021_2113_MOESM5_ESM.tif]

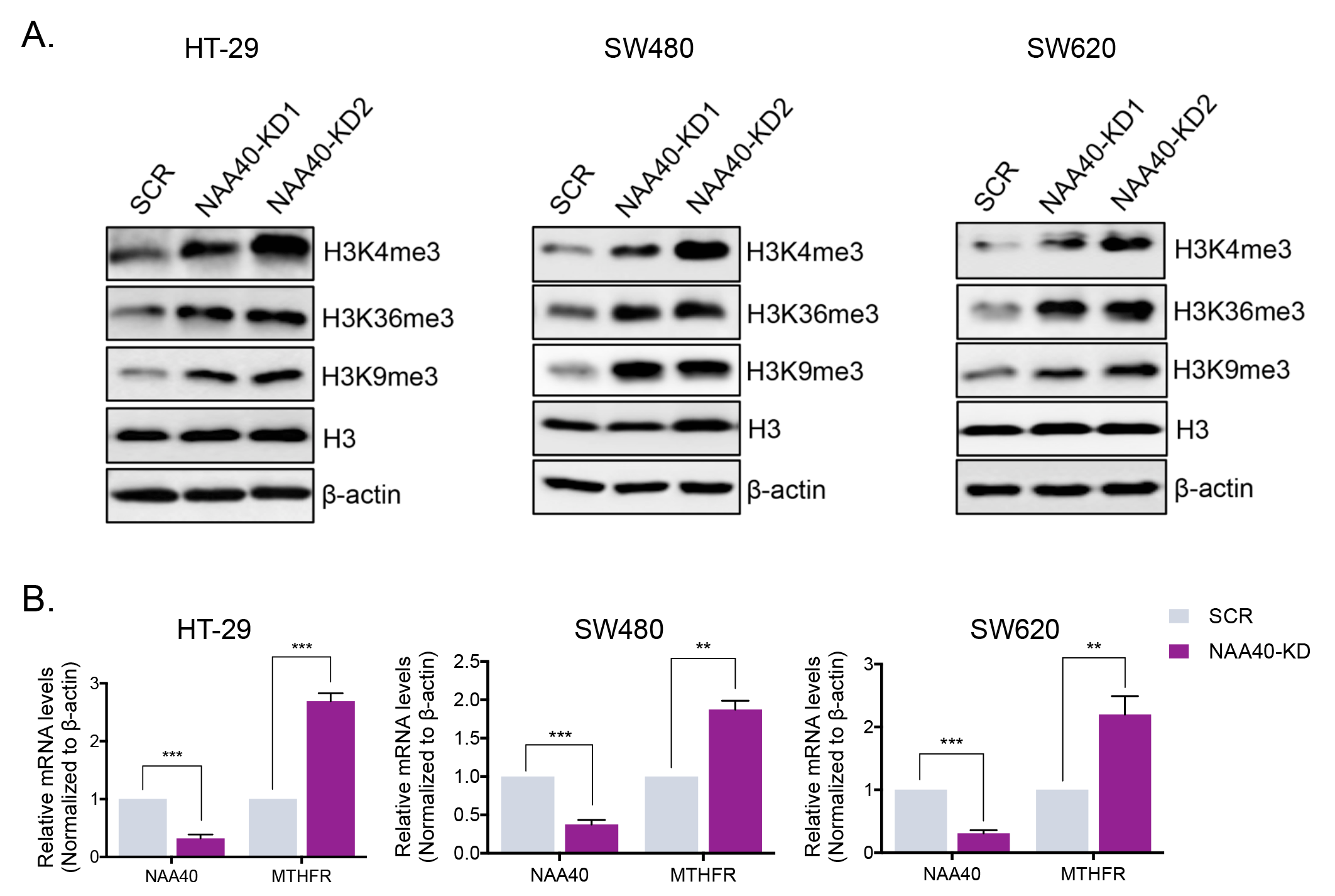

Supplement: Supplementary file 6 — Figure S5 [file 41388_2021_2113_MOESM6_ESM.tif]

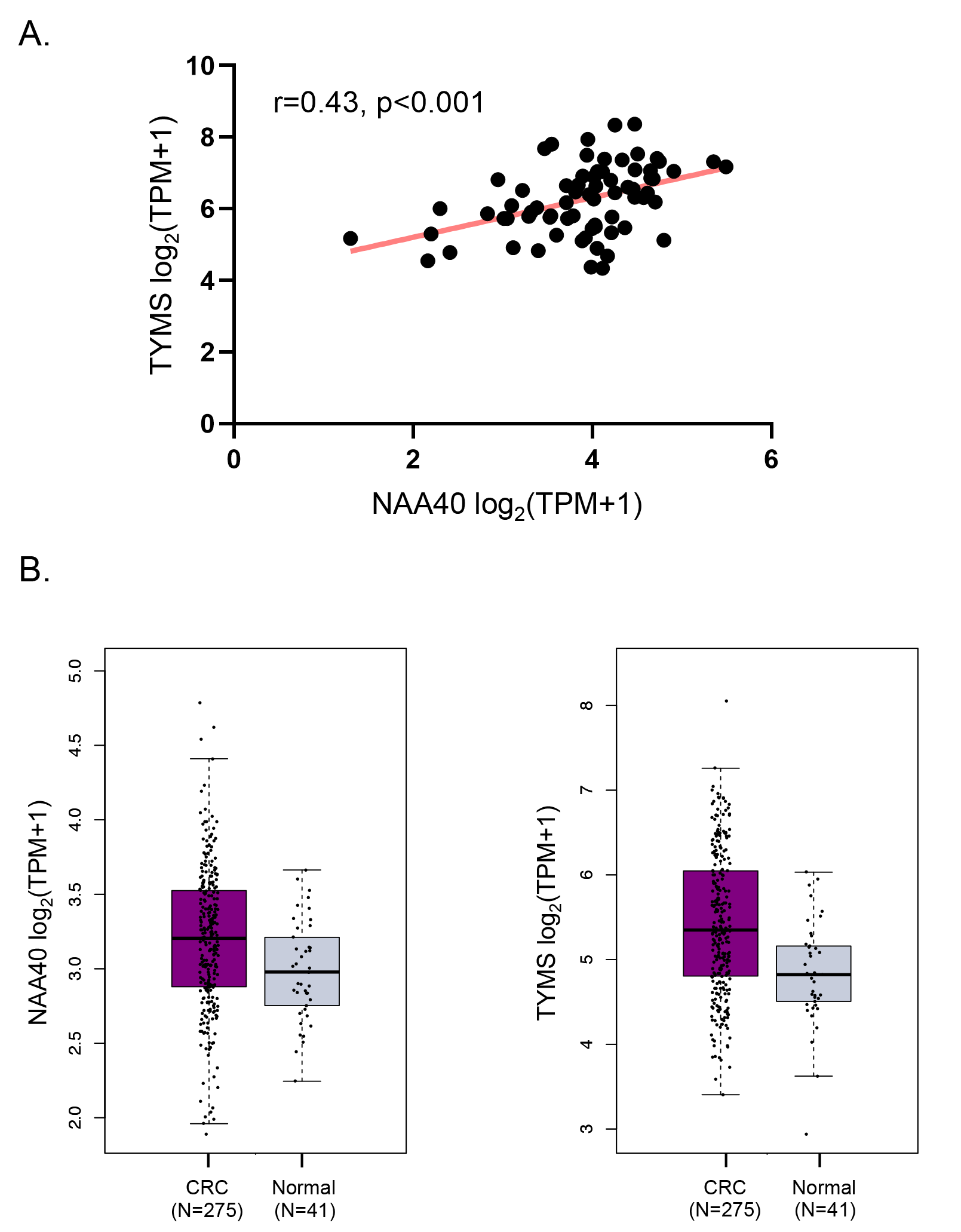

Supplement: Supplementary file 7 — Figure S6 [file 41388_2021_2113_MOESM7_ESM.tif]

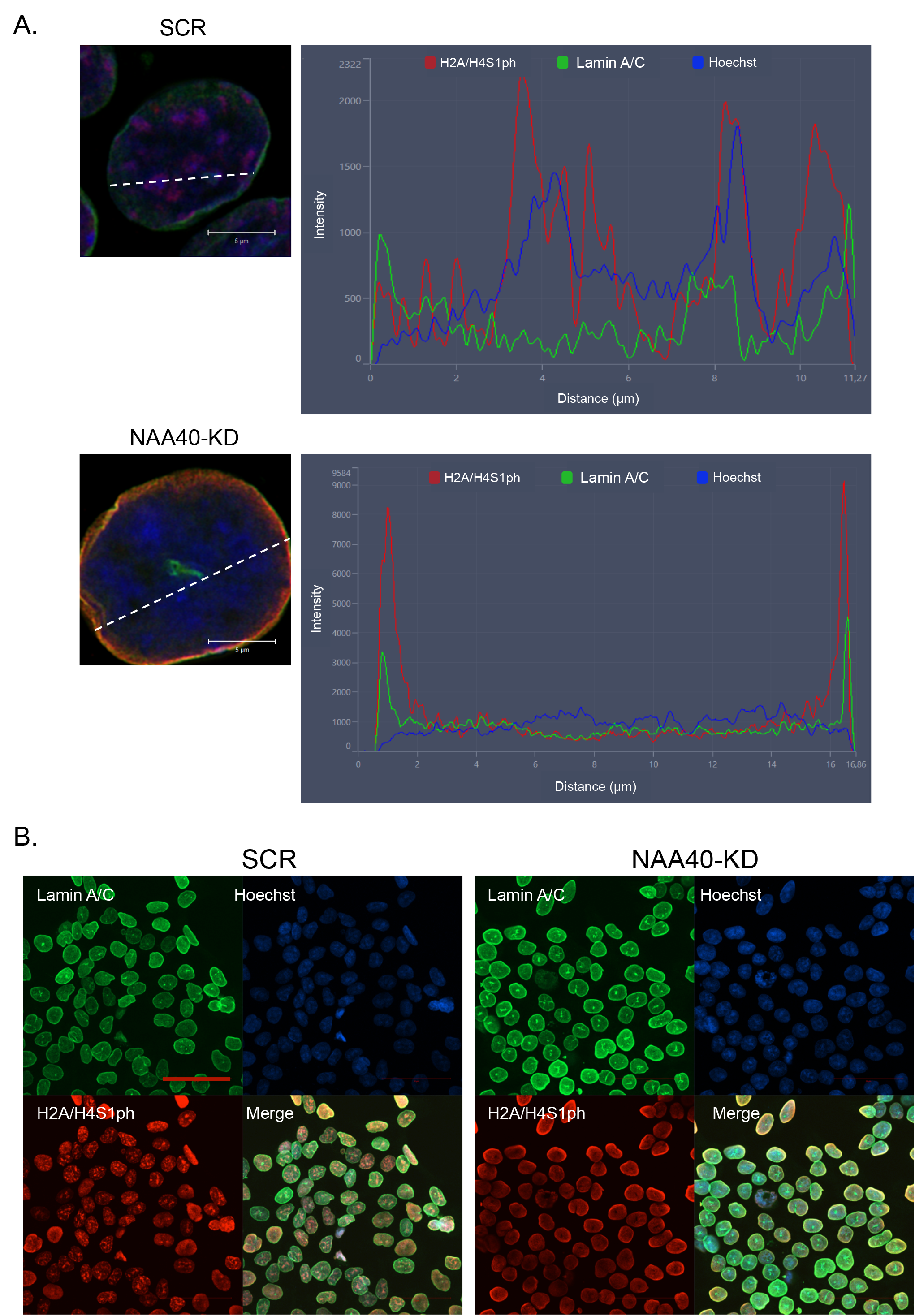

Supplement: Supplementary file 8 — Figure S7 [file 41388_2021_2113_MOESM8_ESM.tif]

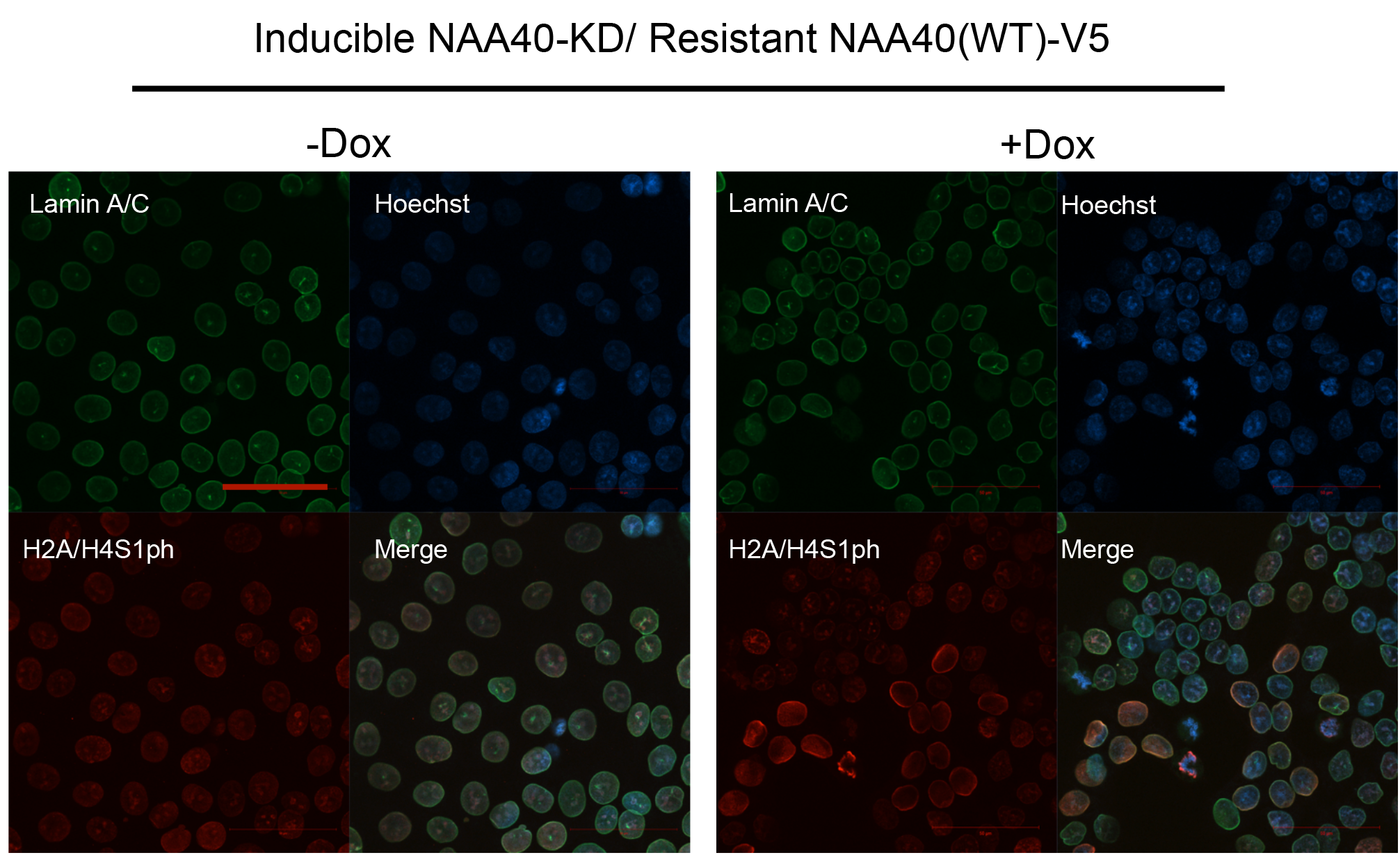

Supplement: Supplementary file 9 — Figure S8 [file 41388_2021_2113_MOESM9_ESM.tif]
