# Supplementary material for: Histone N-terminal acetyltransferase NAA40 links one-carbon metabolism to chemoresistance
Source: Oncogene. 2021 Nov 16;41(4):571–85. doi: 10.1038/s41388-021-02113-9 (PMC8782725; doi:10.1038/s41388-021-02113-9)
Supplement: Supplementary file 10 — Table S1 [file 41388_2021_2113_MOESM10_ESM.docx]

 Table S1. Primers for cloning

| Primers used for the subcloning of NAA40 cDNA from the pOTB7 vector​ | **Forward (5🡪3)**​ | **Reverse (5🡪3)**​ |
| --- | --- | --- |
| Subcloning primer set​ | TGCGGATCCAATGGGGAGAAAGTCAAGC ​ | TTGCTCGAGCCGTGGCAGCAGCCACCACA ​ |
| Primers used for the generation of the Empty vector plasmid​ | **Forward (5🡪3)**​ | **Reverse (5🡪3)**​ |
| Empty vector primer set​ | CGACTGGATCCACGACGCAAGGAATTTCGGACTCTCGAGGACTC ​ | GAGTCCTCGAGAGTCCGAAATTCCTTGCGTCGTGGATCCAGTCG ​ |
| Primers used for the generation of the shRNA Resistant plasmid​ | **Forward (5🡪3)**​ | **Reverse (5🡪3)**​ |
| Rescue primer set A​ | GCACACAGATGAAGAAAGTGATGTTAACAGTA ​ | TACTGTTAACATCACTTTCTTCATCTGTGTGC ​ |
| Rescue primer set B​ | GAAGAAAGTGATGCTGACAGTATTTA AACAC ​ | GTGTTTAAATACTGTCAGCATCACTT TCTTC ​ |
| Rescue primer set C​ | GAAAGTGATGCTGACGGTGTTTAAACACAATCATGG ​ | CCATGATTGTGTTTAAACACCGTCAGCATCACTTTC ​ |
| Primers used for the generation of the catalytic mutant shRNA Resistant plasmid​ | **Forward (5🡪3)**​ | **Reverse (5🡪3)**​ |
| Catalytic mutant primer set ​ | GTCCTGTACTGCTATCAAGTGCAGTTGGAAAGC ​ | GCTTTCCAACTGCACTTGATAGCAGTACAGGAC ​ |
