# Supplementary material for: Histone N-terminal acetyltransferase NAA40 links one-carbon metabolism to chemoresistance
Source: Oncogene. 2021 Nov 16;41(4):571–85. doi: 10.1038/s41388-021-02113-9 (PMC8782725; doi:10.1038/s41388-021-02113-9)
Supplement: Supplementary file 11 — Table S2 [file 41388_2021_2113_MOESM11_ESM.docx]

 Table S2. Primers for real time PCR

| **Gene name**​ | **Forward (5 🡪 3)**​ | **Reverse (5 🡪 3)**​ |
| --- | --- | --- |
| **qRT-PCR**​ | ​ | ​ |
| NAA40 ​ | ATGTAAGCGAGTGTCTGGACT ​ | TGGTTTGCATATTCGTTTTGGTC ​ |
| c-myc​ | CAGCTGCTTAGACGCTGGATTT | ACCGAGTCGTAGTCGAGGTCAT ​ |
| myb​ | GTCCGAAACGTTGGTCTGTT​ | GCCACCTCTCCCTACATTGT​ |
| pola1​ | ATGTGTGCAAAGCTCCTCAC​ | CTTTGGCATGTTGGATCGCT​ |
| TYMS​ | GGGACTTGGGCCCAGTTTAT​ | CTTCTGTCGTCAGGGTTGGT​ |
| MTHFR​ | AGCCGATTTCATCATCACGC ​ | CATGCCTTCACAAAGCGGAA​ |
| MAT1A​ | TGCTGCTGTGTGGTGAGAT​ | GATGTGCTTGATGGTGTCCC​ |
| CTH​ | CCACCCAGAAGGTGATTGA​ | CACGACCAAAATAATGTCTCC​ |
| MMAA​ | TGCATACATCAGGCCATCTC​ | GCTTCATTTGTGGTCCTTGTC​ |
| IFIT1​ | TTCTCCTTGCCCTGAAGCTT​ | TCTGTGAGGACATGTTGGCT​ |
| FOXO4​ | CCATTCCCTGCTATCTCGGA​ | TGCTGTAGGTGTGTAAGGGG​ |
| β-actin​ | AGAGCTACGAGCTGCCTGAC​ | AGCACTGTGTTGGCGTACAG​ |
| **ChIP**​ | ​ | ​ |
| TYMS​ | TCTCTGTGGCTCGACACTTC​ | TGTGAAGACAACACCAAGCA​ |
| Jun ​ | ACTCTGAGCCCTTATCCAGC​ | AAAGAAGGGCCCGACTGTAG​ |
