# Supplementary material for: Histone N-terminal acetyltransferase NAA40 links one-carbon metabolism to chemoresistance
Source: Oncogene. 2021 Nov 16;41(4):571–85. doi: 10.1038/s41388-021-02113-9 (PMC8782725; doi:10.1038/s41388-021-02113-9)
Supplement: Supplementary file 12 — Table S3 [file 41388_2021_2113_MOESM12_ESM.docx]

 Table S3. Antibodies for western blot and IF analysis

| **Antibody name**​ | **Host species**​ | **Dilution**​ | **Catalog #**​ | **Vendor**​ |
| --- | --- | --- | --- | --- |
| H4/H2AS1ph​ | Rabbit​ | 1:1000​ | ab177309​ | Abcam​ |
| H3K4me3​ | Rabbit​ | 1:2000​ | ab8580​ | Abcam​ |
| H3K36me3​ | Rabbit​ | 1:2000​ | ab9050​ | Abcam​ |
| H3K9me3​ | Rabbit​ | 1:1000​ | ab8898​ | Abcam​ |
| H3K27me3​ | Rabbit​ | 1:1000​ | 39156​ | Active motif​ |
| H3K79me3​ | Rabbit​ | 1:1000​ | ab2621​ | Abcam​ |
| H3K79me2​ | Rabbit​ | 1:1000​ | ab3594​ | Abcam​ |
| H3​ | Rabbit​ | 1:4000​ | ab1791​ | Abcam​ |
| H4​ | Rabbit​ | 1:1000​ | 05-805​ | Millipore​ |
| H2A​ | Rabbit​ | 1:1000​ | ab18255​ | Abcam​ |
| β-actin​ | Rabbit​ | 1:1000​ | sc-1616-R​ | Santa Cruz​ |
| V5​ | Rabbit​ | 1:10,000​ | AB3792​ | Millipore​ |
| GAPDH​ | Rabbit​ | 1:2000​ | ab9485​ | Abcam​ |
| Lamin A/C ​ | Mouse​ | 1:1000​ | ab238303​ | Abcam​ |
| NAA40 ​ | Rabbit​ | 1:1000​ | ab106408​ | Abcam​ |
